# Androgen receptor drives divergent chromatin accessibility programs in benign and malignant prostate epithelial cells

**DOI:** 10.1093/nar/gkag891

**Published:** 2026-09-16

**Authors:** Konsta Kukkonen, Ebrahim Afyounian, Hanna Rauhala, Matti Nykter, Alfonso Urbanucci, Leena Latonen, Tapio Visakorpi

**Affiliations:** Faculty of Medicine and Health Technology, Tampere University and Tays Cancer Centre, Tampere University Hospital, 33520 Tampere, Finland; Faculty of Medicine and Health Technology, Tampere University and Tays Cancer Centre, Tampere University Hospital, 33520 Tampere, Finland; Faculty of Medicine and Health Technology, Tampere University and Tays Cancer Centre, Tampere University Hospital, 33520 Tampere, Finland; Faculty of Medicine and Health Technology, Tampere University and Tays Cancer Centre, Tampere University Hospital, 33520 Tampere, Finland; Faculty of Medicine and Health Technology, Tampere University and Tays Cancer Centre, Tampere University Hospital, 33520 Tampere, Finland; Foundation for the Finnish Cancer Institute, 00290 Helsinki, Finland; Department of Tumor Biology, Institute for Cancer Research, Oslo University Hospital, 0424 Oslo, Norway; Institute of Biomedicine, University of Eastern Finland, 70211 Kuopio, Finland; Faculty of Medicine and Health Technology, Tampere University and Tays Cancer Centre, Tampere University Hospital, 33520 Tampere, Finland; Fimlab Laboratories Ltd, Tampere University Hospital, 33520 Tampere, Finland

## Abstract

Androgen receptor (AR) activation alters chromatin structure in prostate cancer (PC) cells, but its impact in nonmalignant prostate epithelial cells (NPEC) remains poorly characterized due to lack of representative models. We leveraged our previously established NPEC (RWPE-1-AR and RWPE-1-Ctrl) and PC (LNCaP-pcDNA3.1 and LNCaP-ARhi) cell models to profile androgen-driven chromatin responses using assay for transposase-accessible chromatin with sequencing (ATAC-seq). In the absence of androgen, NPEC and PC cells exhibited widespread differences in chromatin accessibility. Androgen stimulation amplified these differences, revealing distinct AR-mediated chromatin remodeling between the cell types. Androgen-sensitive regions were enriched for nuclear receptor binding sites (AR and glucocorticoid receptor), with AP-1 motifs more prominent in NPEC and FOXA1 motifs in PC cells.

Comparison with ATAC-seq profiles obtained from clinical samples showed that NPECs resembled benign prostate hyperplasia, while PC cells mirrored untreated and castration-resistant PC, supporting the relevance of our models. Integration of ATAC-seq and mRNA-sequencing (mRNA-seq) identified model-specific AR target genes, including CDKN1C/p57 in NPEC, which may contribute to the previously observed androgen-induced growth arrest in these cells. These findings demonstrate that AR activation reprograms chromatin accessibility in normal prostate epithelium, revealing fundamental regulatory differences compared to the tumor context and underscore the importance of physiologically relevant cell models.

## Graphical Abstract

**Graphical Abstract:**
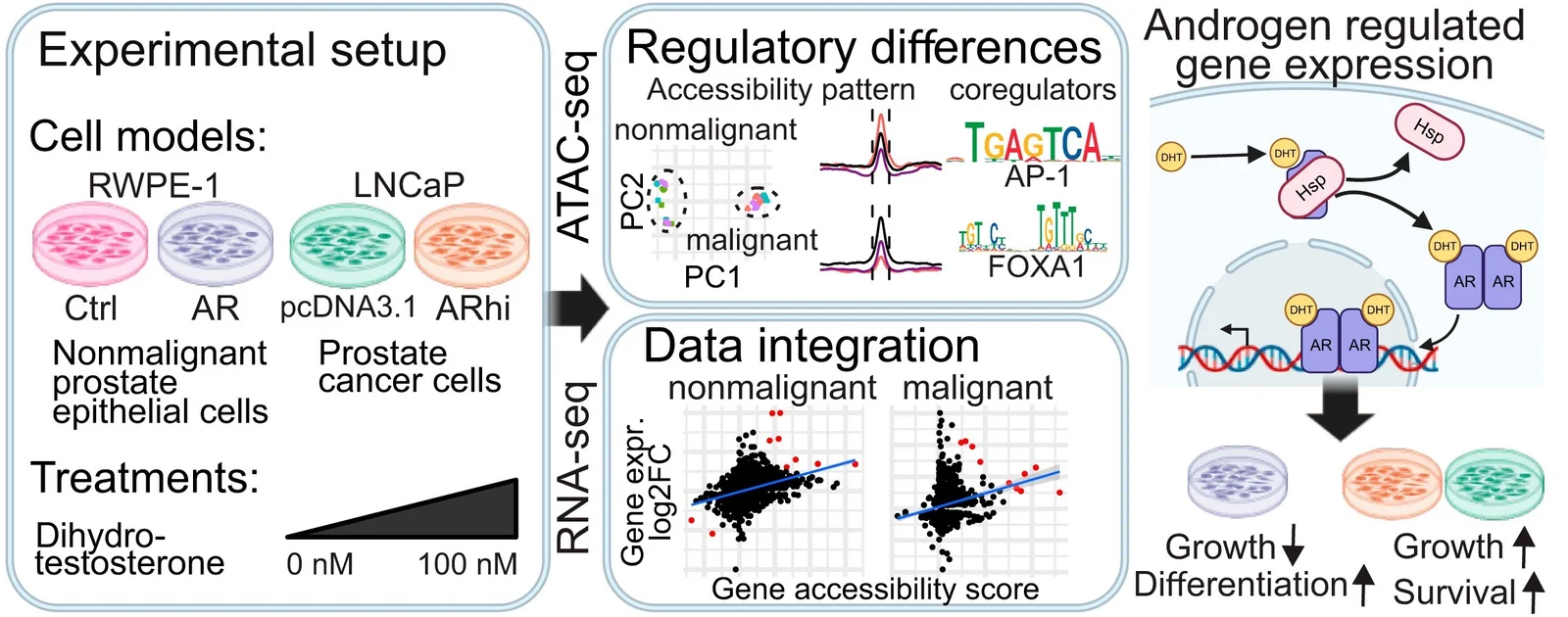

## Introduction

Prostate cancer (PC) is the most common cancer in men in countries with Western lifestyle and the incidence is projected to more than double by 2040 [1, 2]. Despite extensive efforts in characterization of PC biology, targeting the androgen receptor (AR) signaling with androgen deprivation therapy remains the most effective therapeutic option for advanced disease. AR is a nuclear receptor, which translocates to the nucleus upon binding its ligand, dihydrotestosterone (DHT), and regulates the expression of specific target genes. AR signaling inhibitors such as enzalutamide and abiraterone acetate have improved patient survival [3, 4], but resistance eventually emerges through mechanisms similar to those observed for old generation AR-targeting agents such as bicalutamide. Castration-resistant prostate cancer (CRPC) mechanisms include AR overexpression via gene or enhancer amplification, as well as AR gene mutations [4–8].

Most transcription factors (TFs) bind exclusively to open chromatin regions devoid of tightly bound histones. Thus, chromatin accessibility serves as a proxy for TF binding potential and has been extensively studied in both *in vitro* and tissue biopsies by us and others [9–15]. These studies have provided valuable insights into how chromatin accessibility patterns influence gene expression and cellular identity in PC, which is closely linked to AR signaling and therapy resistance [11, 16–18]. However, the effects of AR activation on chromatin in nonmalignant cells remain unexplored, limiting our understanding of cancer-associated chromatin remodeling events.

We previously established and characterized a nonmalignant prostate epithelial cell (NPEC) line (RWPE-1-AR) [19] with ectopic expression of the wild-type AR (wtAR). These cells exhibit benign characteristics, including growth inhibition and differentiation in response to DHT-induced AR activation. In this study, we investigate the effects of androgen stimulation on chromatin accessibility in these nonmalignant cells and compare their responses to those of PC cells expressing either native ligand binding site-mutated (T877A) AR or additional overexpression of wtAR (LNCaP-pcDNA3.1 and LNCaP-ARhi, respectively) [20]. The LNCaP-ARhi model recapitulates features of CRPC with AR overexpression, responding to lower levels of DHT than the control cell line LNCaP-pcDNA3.1 [20, 21]. This comparison enables us to delineate the divergent genomic action of AR in NPEC versus PC cells.

## Materials and methods

### Cell lines and cell culture

RWPE-1-Ctrlc1 and RWPE-1-ARc5 cells (hereafter referred to as RWPE-1-Ctrl and RWPE-1-AR, respectively), as well as LNCaP-pcDNA3.1, and -ARhi, were previously established by our group [19, 20]. RWPE-1-cells were transduced with pWPI-MCS and pWPI-MCS-AR lentiviral vectors, while LNCaP cell lines were generated via transfection with either empty pcDNA3.1 or pcDNA3.1-AR vector. RWPE-1 cells were cultured in Keratinocyte serum-free medium (K-SFM; Gibco/Thermo Fisher Scientific, Waltham, MA, USA), except during the DHT stimulation experiments, for which phenol-red free RPMI-1640 (Lonza, Basel, Switzerland) supplemented with 5% charcoal-stripped fetal bovine serun (FBS) (Sigma–Aldrich/Merck Millipore, Burlington, MA, USA) and 1% L-glutamine was used. LNCaP-pcDNA3.1 and -ARhi were maintained in RPMI-1640 with 10% FBS (Merck Millipore, Burlington, MA, USA), 1% L-glutamine, with 250 µg/ml Geneticin (G418 Sulfate; Gibco/Thermo Fisher Scientific) for selection. For DHT stimulation experiments the same medium as used for RWPE-1 cells was applied, except with 10% charcoal-stripped FBS. All cell lines were subcultured twice weekly. For proteasome inhibition, cells were treated with 5 µM MG132 (Sigma–Aldrich/Merck Millipore) or vehicle dimethyl sulfoxide (DMSO) along with DHT or vehicle (EtOH) for 24 h prior to sample collection. All cell lines were routinely tested for mycoplasma contamination using VenorGem Classic mycoplasma detection kit (Minerva Biolabs GmbH, Berlin, Germany).

### ATAC-seq library preparation and sequencing

Cells were grown for 72 h in hormone-depleted medium and subsequently treated for 2 h with medium supplemented with 0, 1, 10, or 100 nM DHT. Following treatment, cells were harvested, and 5 × 10^4^ cells per sample were used for ATAC-seq library preparation. Library preparation was performed using the Omni-ATAC protocol [22], with the following modifications: the cell centrifugation was conducted at 700 × *g* for 5 min, and transposition was carried out for 45 min in 37°C with agitation at 900 RPM. Library cleanup steps following transposition and amplification were performed using MinElute kit (Qiagen, Hilden, Germany), and final purification was completed using AMPure Xp beads (Beckman Coulter, Brea, CA, USA). Fragment length distribution was assessed using Fragment analyzer with High Sensitivity NGS Fragment Analysis kit (Agilent, Santa Clara, CA, USA). Sequencing was conducted on Novaseq 6000 platform (Illumina, San Diego, CA, USA) by Novogene (Cambridge, UK).

### RNA-sample preparation, reverse transcription and qPCR

RNA-extraction, complementary DNA (cDNA) synthesis, and quantitative real-time PCR, (qPCR) were performed as previously described [19]. Briefly, total RNA was extracted using TRIzol reagent (Invitrogen/Thermo Fisher Scientific) according to manufacturer’s instructions. Reverse transcription was carried out using 1 µg of total RNA per sample with Maxima Reverse Transcriptase (Invitrogen/Thermo Fisher Scientific), following manufacturer’s protocol. qPCR reactions were performed using 2× SYBR Green Master Mix (Thermo Fisher Scientific) on CFX96 Opus Real-Time PCR system (Bio-Rad, Hercules, CA, USA), according to manufacturer guidelines. The oligonucleotides used for qPCR (Merck Millipore) are listed in Supplementary Table S1.

### Western blotting

Western blotting was performed as previously described [19], with the exception that samples were lysed using Triton X-100 lysis buffer and no protein sub-fractionation was performed. Primary-antibodies used were rabbit anti-CDKN1C (HPA002924; Sigma–Aldrich/Merck Millipore) at 1:1000 dilution and rabbit anti-fibrillarin (C13C3; #2639, Cell Signaling Technology, Danvers, MA, USA) at 1:4000 dilution.

### Preprocessing of sequencing data

Quality of raw ATAC-seq reads was assessed using FastQC v0.11.9 (https://www.bioinformatics.babraham.ac.uk/projects/fastqc/), and reads were trimmed using Trim Galore! v.0.6.10 (https://github.com/FelixKrueger/TrimGalore) with the following parameters: –phred33 -q 20 –fastqc –length 36 –paired –retain_unpaired. Trimmed reads were aligned to GRCh38_no_alt_plus_hs38d1_analysis_set reference genome using Bowtie2 v2.4.5 [23] with parameters -X 2000 –very-sensitive -q and written in BAM format using Samtools v1.15.1 [24]. Picard toolkit v2.27.1 (https://broadinstitute.github.io/picard/) was used to assign read groups based on flow cell, fix mate information, remove duplicate reads and reads with MAPQ < 30, and index the BAM files. To retain information of Tn5 transposition activity at both ends of each fragment, BAM files were converted to single-end BED format using Bedtools v2.29.1, facilitating more sensitive and specific peak calling. Peak calling was performed using MACS3 v3.0.0b [25] with the following parameters: -f BED –shift -75 –extsize 150 -g hs –nomodel –nolambda –keep-dup all -q 0.1 –call-summits, resulting in 297 097–788 511 raw peaks per sample. Library quality was assessed using Ataqv v1.3.1 [26]. Key metrics, including the percentage of high-quality autosomal alignments, transcription start site (TSS) enrichment score, and fraction of reads in peaks are presented in Supplementary Table S2.

### Consensus peak calling

To identify reproducible peaks from the raw peak set, we followed the protocol established by The Cancer Genome Atlas (TCGA) consortium [13]. Signal at the reproducible peak regions (*n* = 116 712) was first corrected for background using a method we described previously[12]. The background-corrected data were normalized by trimmed mean of M-values (TMM) method implemented in edgeR v4.4.2 and subsequently converted to counts per million (CPM). Prior to downstream analyses, mitochondrial homologous regions [12] and regions with artificially high signal (ENCODE blacklist) [27] were removed, resulting in final set of 116 126 reproducible peaks. To assess the overall signal distribution across reproducible peak regions, principal component analysis (PCA) was performed using the normalized signal values. The first two principal components were visualized to evaluate sample clustering. For reproducible peak-based analyses, a log_2_(CPM) threshold was determined using the Kneedle algorithm [28]. The first “knee” point at approximately log_2_(CPM) = 2.4, retained ∼90% of the reproducible peaks and was selected as the cutoff (Supplementary Fig. S1A). Peaks passing this threshold were annotated using GENCODE v30 annotation file and the ChIPseeker Bioconductor package [29] to determine their genomic distribution.

### Differential accessibility analysis

For differential accessibility analysis, background-corrected read counts from the reproducible peak set were filtered to retain regions with a minimum read count of 100, resulting in the removal of 15 402 low-signal peaks. The remaining 100 724 peaks were analyzed for differential accessibility using edgeR. Multidimensional scaling of these regions confirmed high consistency across biological replicates (Supplementary Fig. S1B). Significant differentially accessible peaks (DAPs) were defined by and absolute log_2_(fold change) > 1 and false discovery rate (FDR) < 0.05. To identify cell line-associated, DHT-sensitive DAPs, we applied additional criteria: for a peak to be classified as opening in a given cell line, the corresponding cell line had to show significantly higher accessibility than the other cell type(s) in the presence of DHT. For a closing peak, the cell line had to show significantly lower accessibility than the other cell type(s) in the presence of DHT. These DAP sets are referred to throughout the manuscript using the cell line name and the suffix DSO-DAPs for DHT-sensitive opening and DSC-DAPs for DHT-sensitive closing peaks. Downstream analyses were performed only on DS-DAP sets containing more than 50 regions to avoid spurious enrichment results.

### TF binding site enrichment analysis with the curated UniBind dataset

The TF binding data of prostate-specific datasets was obtained from UniBind robust collection, which includes only ChIP-seq peaks containing the corresponding JASPAR motif within the peak region [30, 31]. The data were downloaded on 5 July 2023. For the manually curated UniBind dataset (Fig. 2C), individual peak sets were combined for experiment types with at least two independent datasets derived from the same cell line or tissue under identical experimental conditions, as described in the respective GEO entries or publications. Overlapping peaks from such experiments were merged by intersection, resulting in binding sites supported by at least two independent experiments. Enrichment analysis was performed using GIGGLE [32] and the combo scores were binned to improve feature visibility.

### TF binding site enrichment analysis with all UniBind TFs from prostate datasets, Roadmap Epigenomics and selected publications

For broader enrichment analysis, we included all prostate-specific TF datasets from the UniBind robust collection, the GIGGLE combo scores from different experiments were averaged prior to visualization. For the Roadmap Epigenomics data [33] and the data from Gerhauser, Favero, Risch, and colleagues (2018) [34], we filtered results to display the 30 datasets with the highest enrichment across our DS-DAP sets.

### Motif enrichment analysis

Motif enrichment analysis was performed using XSTREME from the MEME Suite [35, 36] to identify TF binding motifs enriched in the DS-DAPs. The minimum motif width was set to 8 (–minw 8), and HOCOMOCOv13 CORE [37] database was used as the reference for known binding motifs. As background, we used union set of reproducible ATAC-seq peaks from each cell line that passed the signal threshold of log_2_(CPM) > 2.5.

### Footprinting analysis

TF-footprinting analysis was performed using HINT-ATAC[38], a hidden Markov model-based tool optimized for ATAC-seq data. The following parameters were used: rgt-hint footprinting –atac-seq –paired-end –organism = hg38. The consensus peak set across all samples was used as the input for footprinting. Motif matching was performed using default parameters. For differential footprinting analysis the –bc option was used to compare footprint depth between conditions.

### Multimodal data integration

To assess the impact of androgen-driven chromatin regulation on gene expression, we integrated ATAC-seq and RNA-seq data by linking DHT-sensitive DAPs to genes based on proximity to TSS. Specifically, we calculated a gene accessibility score for all differentially expressed (DE) genes within 250 kb of at least one DHT-sensitive DAP from (Fig. 3E). For each gene, accessibility score was computed as the sum of distance-weighted log_2_(fold changes) using the following equation:


\begin{eqnarray*}
{\mathrm{ Accessibility}}\ {\mathrm{ score}} = \ \mathop \sum \limits_{i = 0}^n {{\log }_2}\left( {F{{C}_i}} \right) \times {{e}^{ - \frac{{\left| {{{d}_i}} \right|}}{{50{\mathrm{\ }}000}} + {{e}^{ - 1}}}}
\end{eqnarray*}

where FC_i_ is the accessibility fold change of DAP_i_ and d_i_ is the distance of the DAP_i_ from the gene’s TSS. This equation is a modified version of the decay function described in the ArchR Bioconductor package [39], with slower decay rate (50 000 instead of 5000) to reflect the broader regulatory range of AR, which frequently influences genes located 50–100 kb away [40, 41]. To identify high-confidence AR target candidates, we applied the following filtering criteria: Genes must be significantly DE (FDR < 0.05). Genes regulated in both cell types in the same direction were excluded. Expression change must exceed |log_2_(FC)| > 1. The product of accessibility score and gene expression log_2_(FC) must be >5. Known AR target genes were excluded. Genes must have DESeq2 normalized expression >20. This filtering yielded final list of high-confidence cell-type specific AR target candidates.

### Data visualizations

All visualizations were generated in R. For general plots, including barplots, violinplots, and scatterplots, ggplot2 [42] package was used. Venn diagrams were created using ggVenndiagram [43]. Heatmaps were generated with ComplexHeatmap [44, 45] package while the coverage heatmaps at DS-DAP regions with EnrichedHeatmap [46] package. For coverage heatmaps at the DS-DAP regions, DAPs were categorized based on the lowest DHT concentration comparison in which they were detected (e.g. opening DAPs detected in all comparisons were categorized to “1 versus 0 nM DHT opening”). To improve feature visibility DAP sets smaller than 50 regions were merged with the next DHT comparison category (e.g. “1 versus 0 nM DHT opening DAPs” with only 25 regions was merged with “10 versus 0 nM DHT opening DAPs” to form “1 or 10 versus 0 nM DHT opening DAPs”). For clinical prostate disease subtype-specific peaks (Uusi-Mäkelä *et al*. 2020) and CRPC subtype-specific peak sets (Tang *et al*. 2022), only the mean signal per group is shown for simplicity.

Genome browser track visualizations were created with Gviz [47] Bioconductor package. For coverage heatmaps and genome browser visualizations, genome wide ATAC-seq coverage was converted to CPM-normalized BigWig format with 50 bp resolution using deepTools v3.5.0 [48] and the following parameters: bamCoverage -bs 50 -bl [ENCODE blacklist [27] and ChrM homologous regions [12] –effectiveGenomeSize 2862010578 –normalizeUsing CPM –ignoreForNormalization chrX chrM –ignoreDuplicates.

## Data availability

The source code for the analyses in this manuscript is available on GitHub: https://github.com/NykterLab/KKu_cell_line_ATAC/. Raw sequencing data, BigWig files, background-corrected ATAC-signal tables, and normalized/raw expression tables are deposited in ArrayExpress database: ATAC-seq: E-MTAB-15731 and RNA-seq: E-MTAB-15758. Raw qPCR data and original Western blot films are available on Zenodo: DOI: 10.5281/zenodo.17093003.

## Results

### Chromatin accessibility landscape of NPEC and PC cells mirrors clinical prostate disease subtypes

Since the effects of AR activation on chromatin accessibility in NPEC remain largely uncharacterized, we performed ATAC-seq on our previously described RWPE-1-AR and RWPE-1-Ctrl cell lines [19]. To identify androgen-dependent chromatin accessibility changes we stimulated the cells for 2 hours with vehicle or with 1, 10, or 100 nM DHT. Chromatin accessibility was also profiled in our previously described PC models LNCaP-ARhi and LNCaP-pcDNA3.1 [20].

**Figure 1.**
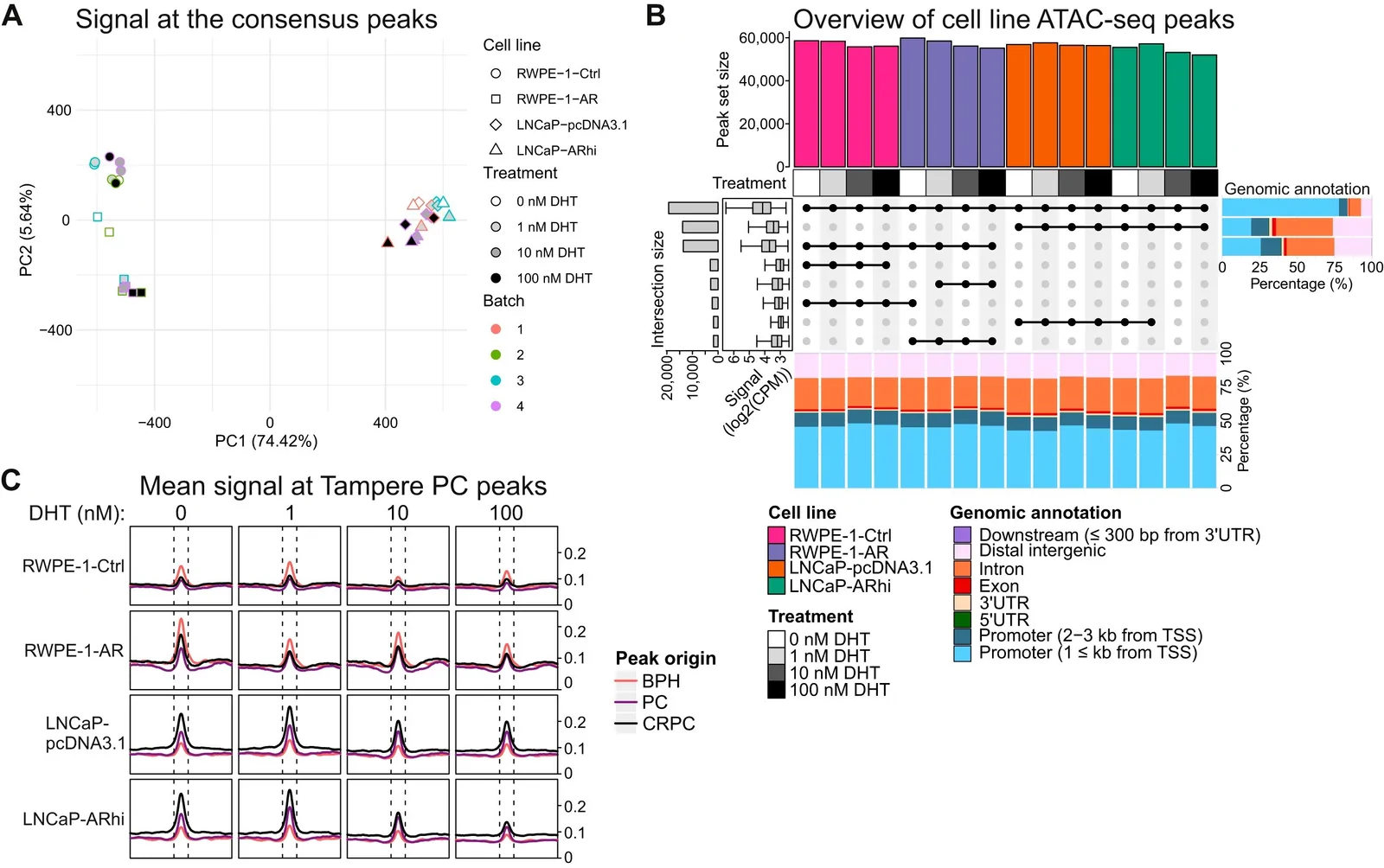
Chromatin accessibility landscape of the dataset. (**A**) PCA representation of the dataset based on normalized ATAC-seq signal at the consensus peaks. (**B**) Upset plot showing consensus peaks in each sample based on data driven log_2_(CPM) threshold of 2.4. Left: Sizes and signal distributions of the eight largest peak set intersections. Right: Genomic annotations of shared and cell line-specific peaks. Below: genomic annotations of consensus peaks. (**C**) Mean ATAC-seq signal at open chromatin regions associated with benign prostate hyperplasia (BPH), untreated PC or CRPC. Each region shown includes the disease-associated peak (500 bp, marked by dashed lines) and ± 1500 bp flanking sequence.

Peak calling identified 116 126 reproducible ATAC-seq peaks across the dataset. PCA of ATAC-seq signal at these peaks revealed clear separation between RWPE-1 and LNCaP samples in the PC1, and the androgen response showing in the AR expressing cell lines mostly in the PC2 (Fig. 1A). The number of reproducible peaks per sample was similar (51,987–59,905), due to TMM normalization. Approximately one-third of the peaks were shared across all studied cell lines and treatment conditions. These peaks were proximal to promoters (mean distance to TSS 2936 bp) and exhibited the highest signal intensity (Fig. 1B). In contrast, peaks shared within a cell type were more distal (mean distance to TSS 14 381 bp for RWPE-1; 20 995 bp for LNCaP) and had lower signal. While 78% of shared peaks were located within the promoter regions (±1 kb from TSS), 57%–64% of cell type-specific peaks were located in distal intergenic or intronic regions. This is consistent with previous reports showing that most TSS regions are open regardless of the gene’s expression status [12, 13], while the cell lineage-specific regulatory regions such as enhancers reside generally outside of the promoters.

To assess whether these chromatin patterns reflect clinical disease states, we compared the cell line data to ATAC-seq profiles from our in-house PC cohort [12] constituting of samples of BPH, untreated PC and CRPC. Indeed RWPE-1 cells showed the highest signal at BPH-associated peaks, while LNCaP cells showed strongest signal at CRPC-associated peaks (Fig. 1C). Furthermore, RWPE-1 derived peaks had the highest overlap with BPH-associated peaks, whereas LNCaP peaks had much greater overlap with PC, and CRPC-associated peaks (Supplementary Fig. S2A–C). These findings support the relevance of our cell models in recapitulating chromatin accessibility patterns of benign and malignant prostate epithelium.

### Androgens change chromatin accessibility of prostate-specific TF binding sites in a cell lineage-specific manner

**Figure 2.**
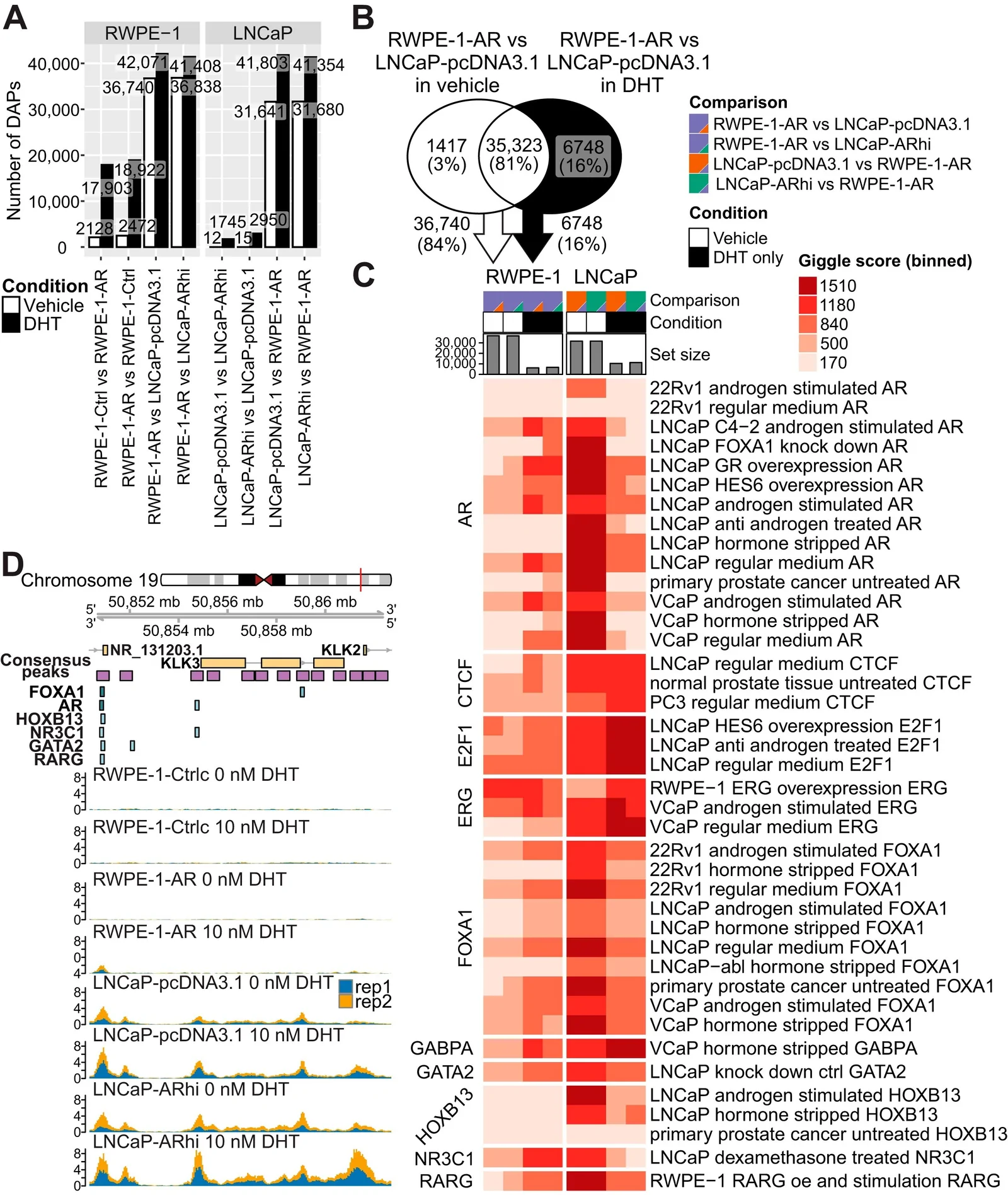
Differences in chromatin accessibility between NPEC and PC cells. (**A**) Number of DAPs between cell lines under baseline conditions (vehicle-treated) and after DHT-treatment (any concentration: 1, 10, or 100 nM). (**B**) Example Venn diagram showing DAPs present under baseline conditions or unique to DHT treatment. Additional Venn diagrams are provided in Supplementary Fig. S3. (**C**) TF binding site enrichment analysis for the DAPs detected in panel (A). Categories correspond to those in panel B: vehicle-treated or DHT-unique DAPs. TF binding site (TFBS) data were manually curated from the prostate-specific datasets of UniBind robust collection (see the ‘Materials and methods’ section). (**D**) Example of ATAC-seq signal at the canonical AR target gene KLK3 [prostate-specific antigen (PSA)] locus. The annotation track shows all consensus ATAC-seq peaks and TFBS from the curated UniBind database overlapping with the region. Signal tracks display aggregate CPM normalized signal for both replicates. The first FOXA1 peak is supported by nine experimental conditions, the second by two, and the first AR peak by 11 conditions in the curated database.

Having established that the cell line models resemble the clinical stages of PC they intended to represent, we next examined AR activation-induced regulatory changes in chromatin. To this end, we performed differential peak analysis across cell lines treated with vehicle only, or by any concentration of DHT (1, 10, or 100 nM; Fig. 2A).

In the absence of DHT, accessibility differences between RWPE-1-AR and RWPE-1-Ctrl, as well as LNCaP-ARhi and LNCaP-pcDNA3.1 were relatively small, as indicated by the low number of DAPs. DHT stimulation markedly increased the number of DAPs, particularly between the RWPE-1-AR and RWPE-1-Ctrl lines, reflecting the AR-negative status of RWPE-1-Ctrl and its lack of responsiveness to DHT. Similarly, the increased number of DAPs between the LNCaP lines is explained by differences in AR expression, which confer greater sensitivity to DHT in LNCaP-ARhi compared to LNCaP-pcDNA3.1 [21].

The largest numbers of DAPs, both in the absence and presence of DHT, was observed between RWPE-1-AR and the LNCaP lines, highlighting substantial differences in regulatory region activity between the nonmalignant and cancer cells. The fact that DHT further accentuates these differences suggests that AR binding patterns differ significantly between the two cell types. Together with our previous findings [9], these results indicate that AR activation is sufficient to drive chromatin accessibility changes in prostate cell lines.

To investigate whether the difference in AR-mediated chromatin accessibility translate into distinct TF occupancy between cancer and nonmalignant cell lines, we performed enrichment analysis using manually curated prostate-specific TFBS datasets from the UniBind database [30, 31] and GIGGLE [32] (see the ‘Materials and methods’ section). For baseline conditions (vehicle treatment), we analyzed all DAPs identified between cell line pairs (e.g. regions more accessible in LNCaP-pcDNA3.1 versus RWPE-1-AR). For DHT comparisons, only DAPs unique to DHT-treated conditions were included (Fig. 2B; additional Venn diagrams in Supplementary Fig. S3A–C).

In the absence of DHT, LNCaP-open DAPs exhibited markedly higher enrichment in 13 of 14 independent AR binding site (ARBS) datasets compared to RWPE-1-AR-open DAPs (Fig. 2C). Similarly, binding sites for AR cofactors such as FOXA1, GATA2, and HOXB13 were more enriched in the LNCaP-open DAPs under baseline conditions. The only TFBS set showing greater enrichment in RWPE-1-AR-open DAPs was ERG ChIP-seq data from RWPE-1-ERG cells.

To determine whether the lower enrichment of ARBS in the RWPE-1-AR-open DAPs reflected a true lack of accessibility, or bias in source data (which predominantly originates from cancer cells), we examined overlap with ARBS from nonmalignant LHSAR cells. These are immortalized prostate epithelial cells (PrEC) ectopically expressing wtAR, hTERT, and large and small t antigen of SV40 virus [49, 50]. RWPE-1-AR-open DAPs under baseline conditions showed the highest overlap with ARBS from LHSAR cells (Supplementary Fig. S3D), suggesting that the lower enrichment observed in the cancer-derived datasets reflects source bias rather than absence of AR binding potential.

Interestingly, DHT stimulation markedly increased enrichment of RWPE-1-AR-open DAPs across ARBS datasets compared to baseline, whereas enrichment of the LNCaP-open DAPs was slightly reduced upon DHT treatment (Fig. 2C). A similar trend was observed for NR3C1 (glucocorticoid receptor, GR) binding sites, with increased enrichment in RWPE-1-AR-open DAPs and reduced in LNCaP-open DAPs, although we note that the expression of *NR3C1* is much lower than that of *AR* in all of the studied cell lines (Supplementary Fig. S3E and F). Changes for FOXA1 and GATA2 were more modest but followed the same pattern. These findings suggest that AR activation induces chromatin accessibility changes at a largely shared pool of TFBS across both cell types, although the sites affected are cell type-specific.

In both absence and presence of DHT, LNCaP-open DAPs were more enriched for E2F1 binding sites than RWPE-1-AR-open DAPs. E2F1 is a positive regulator of cell cycle progression [51] and its increased accessibility in LNCaP cells, despite similar expression levels between cell lines (Supplementary Fig. S3G), may reflect cell cycle deregulation in cancer. This is in line with activation of HALLMARK_E2F_TARGETS signature in LNCaP cell lines in in comparison to RWPE-1-AR after DHT treatment (Supplementary Fig. S3H). Similarly, binding sites for ERG and GABPA derived from *TMPRSS2-ERG* fusion-positive VCaP cells were more accessible in LNCaP than in RWPE-1-AR, particularly under DHT stimulation.

Chromatin accessibility can directly affect gene expression. In our models, this is exemplified by the *KLK3* locus. In the LNCaP cell lines, which express KLK3 (PSA), several accessible regions were detected at the promoter and gene body, whereas none were observed in RWPE-1-AR (Fig. 2D). This is consistent with our previous results that *KLK3* messenger RNA (mRNA) is undetectable in RWPE-1-AR, despite expression of several other canonical AR target genes [19].

### AR activation changes chromatin accessibility landscape in androgen-naïve cells

**Figure 3.**
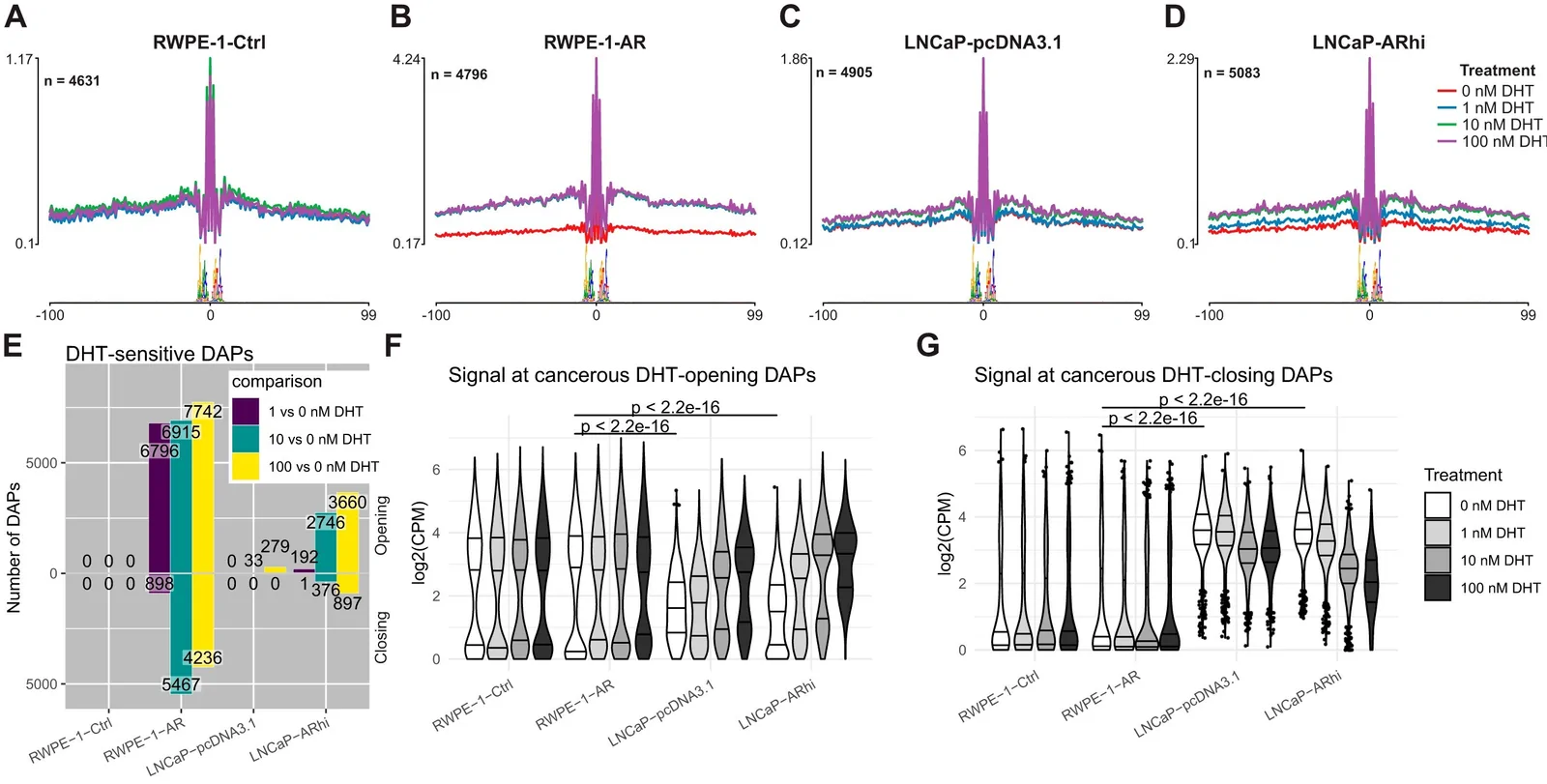
DHT-sensitive DAPs in AR-expressing cells. (**A–D**) Accessibility footprints at sites containing AR motif in RWPE-1-Ctrl (**A**), RWPE-1-AR (**B**), LNCaP-pcDNA3.1 (**C**), and LNCaP-ARhi (**D**) cells under different DHT conditions. Note that partially different footprint regions were selected for analysis. (**E**) Number of DHT-sensitive DAPs (both opening and closing) in each cell line. (**F, G**) TMM-CPM normalized ATAC-seq signal strength at DAPs identified as opening [panel (F); padj < 0.05 and log_2_(fold change) > 1] or closing [panel (G); padj < 0.05 and log_2_(fold change) < −1] only in cancer cell lines. Statistical tests shown in panels (F) and (G) are based on two-tailed Mann–Whitney U tests. Horizontal lines indicate 0.25, 0.5, and 0.75 quartiles; values exceeding 1.5× the interquartile range above or below are shown as outliers.

To further examine the effects of AR activation on chromatin accessibility, we performed TF footprinting analysis to the ATAC-seq data using hidden Markov model-based footprinting tool HINT-ATAC [38]. Differential footprinting revealed that increasing DHT concentrations enhanced AR footprint depth in AR-expressing cell lines, whereas no change was observed in AR-negative RWPE-1-Ctrl cells (Fig. 3A–D). The greatest difference in footprint depth between DHT and vehicle treatments occurred in RWPE-1-AR cells, although the specific regions selected by the footprinting algorithm varied slightly between cell lines. We also noted that AR expression level influenced chromatin response to DHT: LNCaP-ARhi exhibited increased footprint depth at 1 nM DHT, whereas LNCaP-pcDNA3.1 required 10 nM DHT to show a similar effect. These findings align with our previous results demonstrating that LNCaP-ARhi cells have more ARBS than LNCaP-pcDNA3.1 at 1 nM DHT [21].

To dissect accessibility changes induced by increasing DHT concentrations, we compared samples treated with 1, 10, or 100 nM DHT against vehicle-treated controls within each cell line (Fig. 3E). The number of DHT-sensitive DAPs increased with higher DHT concentrations in all AR-positive cell lines. Interestingly, RWPE-1-AR cells exhibited more DAPs than LNCaP lines, and the number of DAPs correlated with AR expression level. These results demonstrate that AR activation drives substantial chromatin remodeling not only in cancer cells as previous shown [9, 14, 52], but also in NPEC.

To assess the cell line specificity of DHT-sensitive DAPs, we examined the normalized ATAC-signal across these regions, excluding DAPs shared between cell types (Fig. 3F and G, and Supplementary Fig. S4A and B). For clarity, we refer to RWPE-1-AR derived DAPs as “nonmalignant DHT-sensitive DAPs” and those from LNCaP lines as “cancerous DHT-sensitive DAPs”.

Simple overlap-based filtering was insufficient to capture cell type-specific regulation, as many DAPs showed baseline accessibility differences independent of DHT exposure. For example, vehicle-treated RWPE-1 cells exhibited significantly higher median accessibility at cancerous DHT-sensitive opening peaks than the LNCaP lines (Fig. 3F), suggesting that large proportion of these regions were already open in the nonmalignant cells. Conversely, cancerous DHT-closing DAPs showed consistently lower signal in RWPE-1 cells than in LNCaPs (Fig. 3G), indicating no AR regulation in the nonmalignant context. Similarly, nonmalignant DHT-opening DAPs were more accessible in the LNCaP cells even without DHT, but DHT stimulation increased accessibility in RWPE-1-AR cells beyond LNCaP levels (Supplementary Fig. S4A). For nonmalignant DHT-closing DAPs, all LNCaP samples had lower accessibility than RWPE-1-AR cells across conditions (Supplementary Fig. S4B).

### DHT-sensitive regions of NPEC are enriched with binding motifs of noncanonical AR binding partners

**Figure 4.**
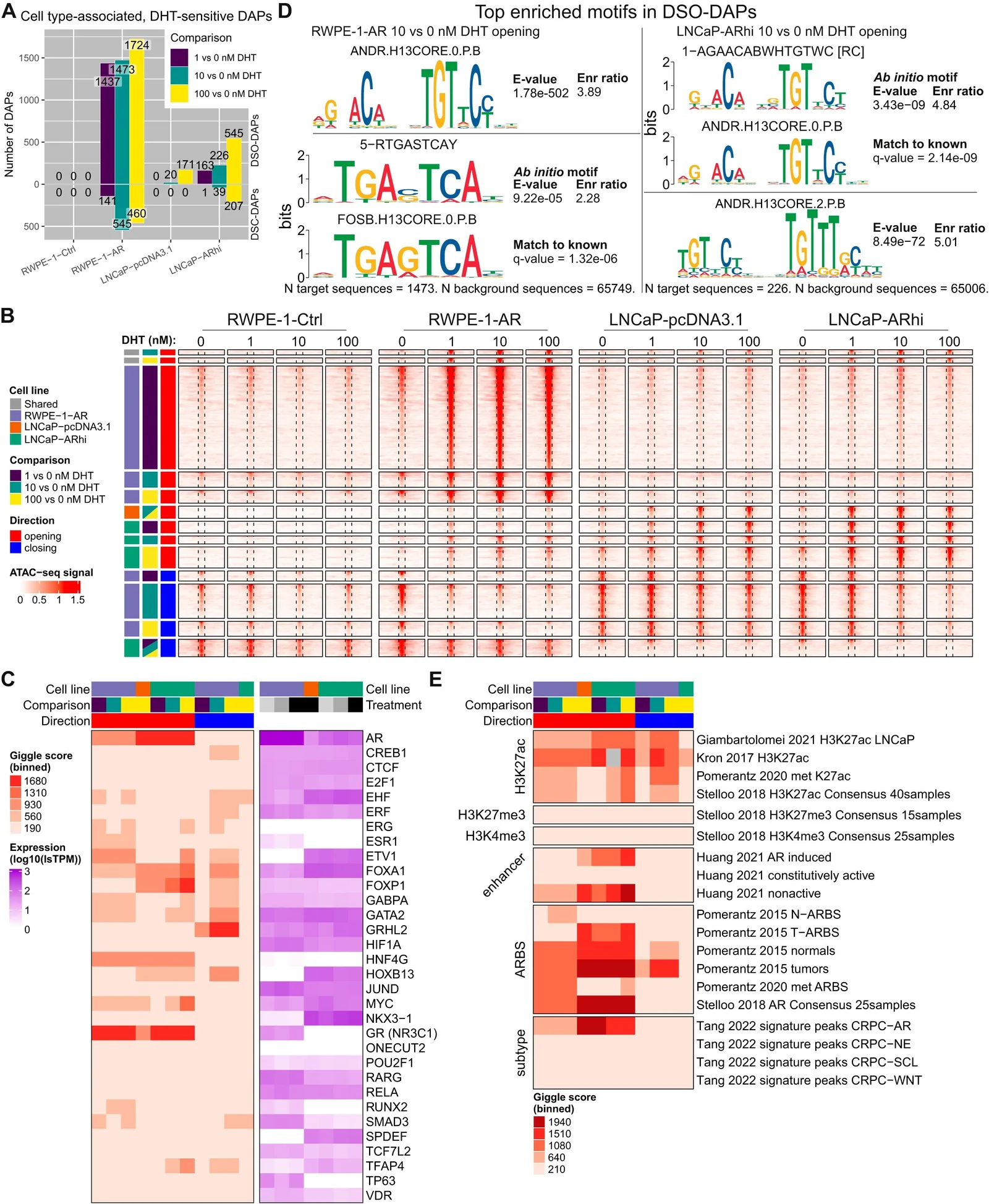
Cell line-associated, DHT-sensitive DAPs. (**A**) Number of DS-DAPs in each sample. (**B**) CPM-normalized ATAC-seq signal at DS-DAPs. Each DAP is shown only once, with DAPs detected in multiple comparisons assigned to the set corresponding to the lowest DHT concentration. Sets smaller than 50 regions were merged with the next DHT comparison category, resulting in split comparisons for some DAP categories. Normalized signal is displayed for the DAP region (500 bp, marked by the dashed lines) and ±1500 bp flanking sequence. (**C**) Enrichment of DSO- and DSC-DAPs in prostate sample-derived TFBS from the UniBind database. TF expression was quantitated by mRNA-seq. (**D**) Top enriched motifs in 10 versus 0 nM DHT DSO-DAPs in RWPE-1-AR and LNCaP-ARhi. The top-ranked motif from RWPE-1-AR is the ARE motif (ANDR.H13CORE.0.P.B), whereas in LNCaP-ARhi it is an *ab initio* motif similar to ARE. The comparison between the *ab initio* motif and ARE is shown. The second-ranked motif in RWPE-1-AR resembles FOSB binding motif (shown in the figure), while in LNCaP-ARhi it is an ARE/FOX motif. (**E**) Enrichment of DS-DAPs in chromatin features from selected publications.

To identify DHT-sensitive regions contributing to the contrasting cellular responses to AR activation in nonmalignant and cancer cells, we applied filtering based on inter-cell line DAPs (Fig. 2A; see the ‘Materials and methods’ section). These filtered regions are referred to as cell line-associated, DHT-sensitive DAPs (DS-DAPs). The number of DS-DAPs in each comparison are presented in Fig. 4A. Coverage heatmaps and ATAC-seq signal distributions confirmed high cell line specificity of these DS-DAPs (Fig. 4B and Supplementary Fig. S4C–F).

Genomic distribution of the DS-DAPs across cell lines was relatively uniform. RWPE-1-AR DSO-DAPs overlapped slightly more with promoter regions, whereas LNCaP DSO-DAPs were enriched in intronic and distal intergenic regions (Supplementary Fig. S4G). For DSC-DAPs, both RWPE-1-AR and LNCaP-ARhi showed an increased proportion of promoter annotations with rising DHT concentrations, while LNCaP-ARhi exhibited a shift from intronic to distal to intergenic regions.

To assess TF binding potential within DS-DAPs, we performed enrichment analysis using prostate-specific TFBS datasets from the UniBind robust collection [30, 31]. RWPE-1-AR DSO-DAPs showed the highest enrichment for GR binding sites, whereas LNCaP DSO-DAPs were strongly enriched for both AR and GR binding sites (Fig. 4C). Overlap analysis revealed that nearly all DSO-DAPs from all cell lines contained ARBS, and up to 60% of RWPE-1-AR DSO-DAPs also overlapped GRBS (Supplementary Fig. S4H–P). RWPE-1-AR DSO-DAPs exhibited weak enrichment for EHF, ESR1 (encoding estrogen receptor 1), RUNX2, and SMAD3 binding sites, which were absent in LNCaP-derived DSO-DAPs. Conversely, RWPE-1-AR DSC-DAPs were most enriched in GRHL2 binding sites, along with FOXA1, GATA2, and HOXB13 (Supplementary Fig. S4N–P), which are pioneering factors previously implicated in prostate lineage specification and PC progression [53, 54]. Notably, HOXB13 expression was very low in RWPE-1-AR, whereas other DSC-DAP enriched TFs were more prominently expressed.

In contrast, LNCaP-ARhi DSO-DAPs displayed strong enrichment to forkhead box (FOX) family binding sites (FOXA1, FOXP1), as well as HOXB13 and TFAP4 binding sites (Fig. 4C). Nearly all LNCaP-ARhi DSO-DAPs containing ARBS also harbored FOXA1 or HOXB13 sites, whereas this proportion was approximately two-thirds for RWPE-1-AR (Supplementary Fig. S4H–M).

To complement ChIP-seq-based enrichment, we performed motif analysis using XSTREME [35, 36] and HOCOMOCO v13 CORE database [37]. In RWPE-1-AR DSO-DAPs, androgen response element (ARE) was the most enriched motif (∼84% of regions; enrichment ratio 3.9–4.1 over background) across all comparisons (1, 10, and 100 nM DHT versus vehicle) (Fig. 4D and Supplementary Fig. S5A–C). Additionally, AP-1 factor binding motifs (e.g. FOSB, JUNB, FOSL1) were significantly enriched (up to ∼2.3 fold over background; present in 45% of regions; Fig. 4D, and Supplementary Fig. S5A–C and I). Interestingly, AP-1 components JUN (c-JUN) and FOS (c-FOS) are induced by DHT in RWPE-1-AR cells (Supplementary Fig. S5J and K) potentially explaining increased accessibility at AP-1 motifs.

In LNCaP-pcDNA3.1 DSO-DAPs motifs resembling ARE/FOX binding motif (AR half-site and FOX motif) were the most enriched followed by FOXA2 motif (Supplementary Fig. S5D). LNCaP-ARhi DSO-DAPs showed enrichment for steroid receptor-like *ab initio* motif or progesterone receptor response element (very similar to ARE) and ARE/FOX motifs (Fig. 4D, and Supplementary Fig. S5E, F, and L). RWPE-1-AR DSC-DAPs lacked strong motif enrichment at 1 versus 0 nM DHT but showed GRHL1/2-like motifs at 10 and 100 versus 0 nM DHT, consistent with upregulation of *GRHL1* and *GRHL2* transcripts under these conditions (Supplementary Fig. S5G, H, and M–P). LNCaP-ARhi DSC-DAPs did not yield motifs with convincing similarity to known TF binding motifs.

In summary, the motif analysis corroborates TFBS enrichment results and further suggests that RWPE-1-AR DSO-DAPs are enriched at sites co-bound by lineage-differentiating factors such as AP-1.

### DHT-sensitive regions of NPEC are enriched with regions mediating cell differentiation

We next compared RWPE-1-AR and LNCaP-ARhi DS-DAPs with PC-derived H3K27ac profiles [53, 55–57], as this histone mark is widely used as a proxy for active enhancers (Fig. 4E). LNCaP-ARhi DSO-DAPs exhibited markedly higher enrichment of H3K27ac sites compared to RWPE-1-AR DSO-DAPs. Interestingly, RWPE-1-AR DSC-DAPs also showed strong enrichment for H3K27ac marks, suggesting redistribution of enhancer activity following AR activation. LNCaP DSO-DAPs were also more enriched to STARR-seq-validated AR-induced and nonactive enhancers [58], whereas the RWPE-1-AR DSO-DAPs displayed only moderate enrichment to the latter set of regions. These results indicate that LNCaP-ARhi DSO-DAPs are more closely associated with cancer-specific enhancer activity.

When examining ARBS datasets from studies by Pomerantz *et al*. (2015), Pomerantz *et al*. (2020), and Stelloo *et al*. (2018) [50, 53, 55], RWPE-1-AR DS-DAPs showed a trend toward enrichment in normal tissue-specific ARBS. Specifically, DSO-DAPs were slightly more enriched in N-ARBS, while DSC-DAPs were more enriched in Pomerantz 2015 tumor ARBS. In contrast, LNCaP DS-DAPs exhibited a strong trend toward tumor tissue-specific ARBS, with DSO-DAPs enriched in tumor-specific ARBS datasets, including T-ARBS, Pomerantz 2015 tumors, and Stelloo 2018 tumor consensus (Fig. 4E). Importantly, N-ARBS represents a functionally relevant subset of Pomerantz 2015 normal, defined as ARBS located within 50 kb of a gene with higher expression in normal tissues than in tumors. Similarly, T-ARBS is a specific subset of Pomerantz 2015 tumor ARBS associated with genes more highly expressed in tumors.

Additionally, LNCaP DSO-DAPs showed higher enrichment for ATAC-seq peaks from the AR subtype (CRPC driven by AR) identified by Tang *et al*. (2022) [16]. Because TFBS enrichment analysis focuses only on AR-regulated regions, we next assessed whether the global chromatin landscape of LNCaP cells most closely resembles signature from AR subtype. To this end, we examined the average ATAC-seq signal at peaks specific to different CRPC subtypes defined by Tang, Xu, Wang and colleagues (2022). The signal enrichment analysis revealed that the global chromatin accessibility pattern of LNCaP cells indeed aligns most strongly with the AR subtype (Supplementary Fig. S6A). RWPE-1 lines exhibited the greatest similarity to the stem cell-like (SCL) signature, consistent with the less differentiated gene expression signature of RWPE-1 in comparison to LNCaP and expression of several key TFs of the subtype such as AP-1 complex members, and TP63 [19]. In RWPE-1-AR cells, DHT stimulation slightly reduced accessibility at SCL signature peaks while simultaneously modestly increasing accessibility at AR subtype-specific regions.

To determine whether RWPE-1-AR chromatin responses would align more closely with normal cell types, we performed enrichment analysis against chromatin states from the Roadmap Epigenomics project and prostate cell line data from Gerhauser *et al*. (2018) [34]. RWPE-1-AR DSO-DAPs were enriched for enhancers from diverse cell types, including epithelial cells (prostate, mammary, intestinal, and epidermal), muscle, brain, embryonic and mesenchymal stem cells as well as cancer cell lines such as A549 (lung cancer), HeLa (cervical cancer) and PC-3 (AR-negative PC; Supplementary Fig. S6B). Interestingly, RWPE-1-AR DSC-DAPs from 10 and 100 versus 0 nM DHT comparisons were also enriched for enhancers from epithelial and cancer cell types. In contrast, LNCaP DSO-DAPs were strongly enriched for LNCaP-derived enhancers and transcribed regions, as well as VCaP derived promoters, while LNCaP-ARhi DSC-DAPs (100 versus 0 nM DHT) showed enrichment for enhancer regions from several epithelial cell types including normal PrEC. These findings indicate that androgen regulation in LNCaP cells is largely unique to AR-positive PC, whereas RWPE-1-AR responses most resemble regulatory patterns observed in normal cell types.

### Multimodal data integration identifies CDKNC1/p57 as a key cell cycle repressor in benign prostate cells

**Figure 5.**
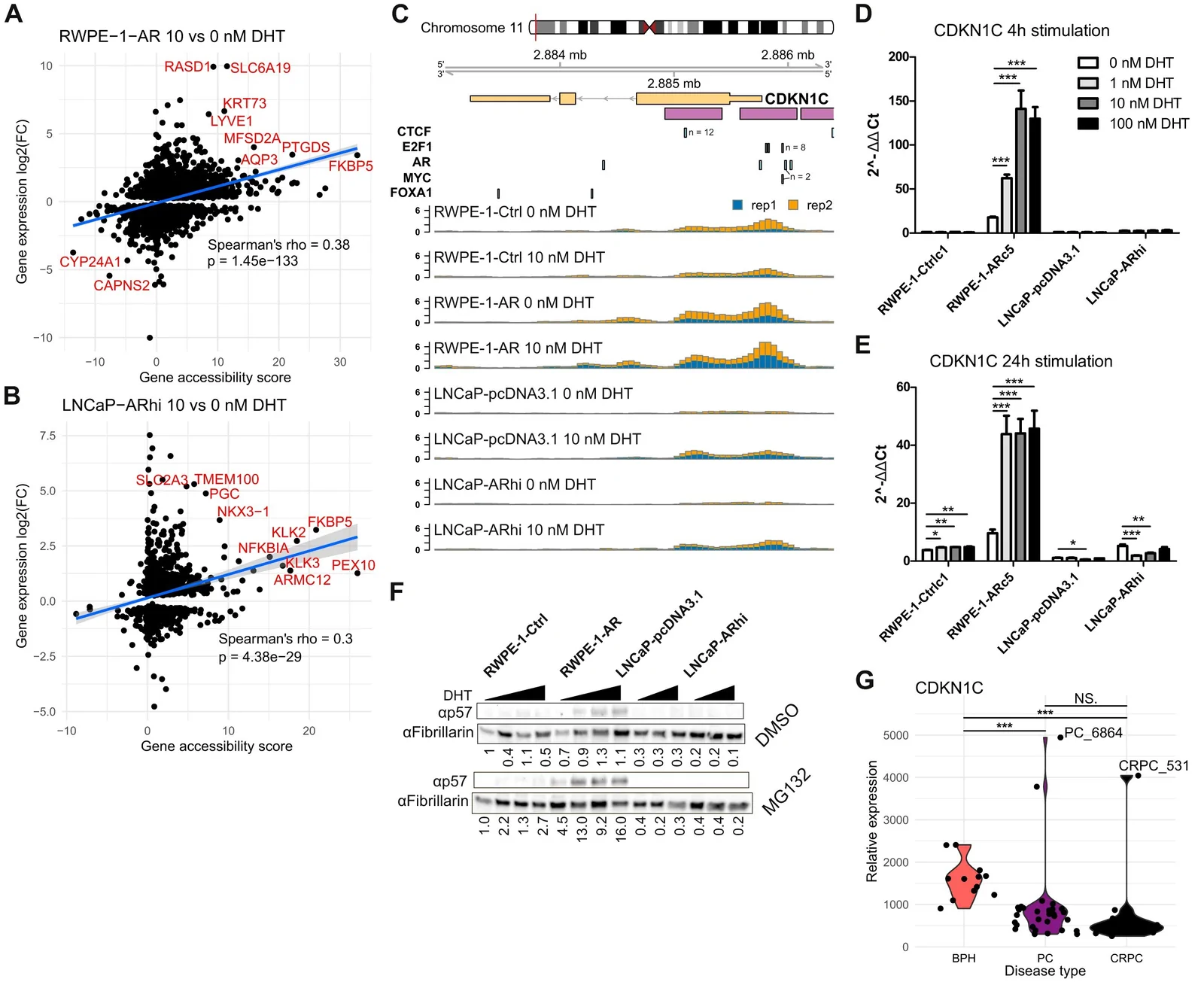
Integration of RNA-seq and ATAC-seq. (**A, B**) Gene expression log_2_(fold change) of significantly DE genes (FDR < 0.05) plotted against accessibility score in RWPE-1-AR (**A**), and LNCaP-ARhi (**B**) at 10 versus 0 nM DHT comparison. Spearman’s ρ and *P*-values for the significance (asymptotic t-approximation) are shown. (**C**) Genomic context of *CDKN1C*. Three ATAC-seq consensus peaks (lilac) and TFBS from independent prostate specific experiments in the UniBind robust collection (light blue) are indicated. Normalized ATAC-seq signal tracks for 0 and 10 nM DHT in each cell line are shown below. (**D, E**) Validation of *CDKN1C* induction with qPCR after 4 h (**D**) or 24 h (**E**) of DHT stimulation. Data represents three biological replicates per condition. (F) Validation of p57 protein induction after 24 h stimulation in the presence of vehicle (DMSO) or 5 µM proteosome inhibitor MG132. (**G**) *CDKN1C* mRNA expression in 12 BPH, 30 PC, and 13 CRPC samples. *P*-values are from Tukey’s *post-hoc* analysis following one-way analysis of variance (ANOVA).

To evaluate the functional impact of androgen-driven chromatin remodeling on gene expression, we integrated ATAC-seq and mRNA-seq by linking DHT-sensitive DAPs to genes within 250 kb of their TSS. For each gene, we calculated an accessibility score (see the ‘Materials and methods’ section) as the sum of distance weighted log_2_(fold changes) for all associated DAPs. Visualization of gene expression log_2_(fold change) versus accessibility score showed weak-to-moderate correlation in RWPE-1-AR (range of Spearman’s rho 0.38–0.46) (10 versus 0 nM DHT in Fig. 5A; rest of the comparisons in Supplementary Fig. S7A and B). In LNCaP-ARhi cells, correlations were weaker (Fig. 5B and Supplementary Fig. S7C).

Accessibility scores were skewed towards positive values, reflecting the predominance of opening DAPs over closing DAPs and equal weighting of positive and negative accessibility changes. Correlation was strongest for upregulated genes, although some downregulated genes also exhibited positive accessibility scores, indicating that certain regions may act as repressive elements.

To prioritize candidate AR target genes, we ranked DE genes by the product of accessibility score and expression log_2_(fold change). Top-ranked genes included canonical AR targets, such as *FKBP5* in both cell lines, *ZBTB16* in RWPE-1-AR and *KLK2, KLK3*, and *NKX3-1* in LNCaP-ARhi (Fig. 5A and B, and Supplementary Fig. S7A–C). After applying stringent filtering criteria including removal of mutually regulated genes and known AR targets (see the ‘Materials and methods’ section), we identified 78–205 genes RWPE-1-AR-specific genes across comparisons and 1–15 LNCaP-ARhi-specific genes. Full lists are provided in Supplementary Tables S3–S7.

Among RWPE-1-AR specific genes, *CDKN1C* (encoding p57, a cyclin-dependent kinase inhibitor) emerged as a top candidate. CDKN1C arrests cell cycle progression in G1 checkpoint and has been implicated in early senescence in hepatocellular carcinoma [59]. In PC, *CDKN1C* gene is frequently silenced by promoter methylation [60, 61]. In our dataset the *CDKN1C* locus contained reproducible ATAC-seq at the promoter where the accessibility increased in RWPE-1-AR cells following stimulation with 10 nM DHT, but not in the other cell lines (Fig. 5C). Broader genomic context, including upstream and downstream RWPE-1-AR DSO-DAPs and overlapping ARBS, is shown in Supplementary Fig. S7D.

We validated *CDKN1C* induction at the mRNA level by qPCR after 4 and 24 h of DHT treatment, and at the protein level after 24 h, with enhanced detection in the presence of the proteosome inhibitor MG132 (Fig. 5D–F). p57 protein expression was highest in RWPE-1-AR cells among all studied lines, consistent with greater accessibility at the *CDKN1C* locus compared to RWPE-1-Ctrl even in the absence of DHT. In our in-house cohort, the expression of *CDKN1C* was significantly higher in BPH than in PC, or CRPC samples, despite occasional outliers (Fig. 5G) [62, 63]. These findings align with TCGA-PRAD data showing reduced *CDKN1C* expression in tumors compared to normal tissue [64, 65] (Supplementary Fig. S7E). However, the expression level of *CDKN1C* in the TCGA-PRAD cohort does not differentiate between aggressive and indolent cancer cases (Supplementary Fig. S7F).

## Discussion

The chromatin landscape of PC has been extensively studied using clinical samples [11–13], patient-derived organoids [16], and cell lines to characterize influence of AR activation on chromatin [9, 14, 52]. However, similar analyses in NPEC have been lacking. In this study, we profiled open chromatin regions in AR-positive NPECs and PC cells under androgen-deprived and androgen-stimulated conditions. Our results reveal that >50% of accessible peaks are mutually exclusive between NPEC and PC cells in the absence of DHT, and this proportion increases to ∼70% following DHT stimulation. These findings indicate a divergent genomic response to AR activation in nonmalignant versus cancerous contexts, which likely underpins their contrasting transcriptional programs.

We observed increased binding potential for several steroid receptors in RWPE-1-AR-open regions following DHT stimulation (Fig. 2C). The enrichment of GRBS in RWPE-1-AR DHT-unique regions may reflect AR binding to sites that overlap GRBS, given the similarity between AR and GR consensus sequences and the higher expression of AR relative to GR in these cells (expression ratio 20–35:1 across different DHT concentrations). Alternatively, this observation aligns with the assisted TF loading model [66], wherein AR binding facilitates chromatin remodeling and creates favorable conditions for GR recruitment as recently demonstrated in PC cells [67]. Similarly, enrichment of FOXA1 and GATA2 binding sites in RWPE-AR DHT-unique regions suggests that AR binding may stabilize pioneering factor occupancy, consistent with recent findings in LNCaP [15].

In LNCaP DHT-unique DAPs, enrichment for E2F1 binding sites was particularly strong. E2F1 is a TF that promotes cell cycle progression [51]. This enrichment, despite similar E2F1 expression levels between cell lines, suggests that AR activation selectively opens E2F1-bound chromatin in cancer cells, supporting their proliferative phenotype [68]. In contrast, AR activation in NPEC exerts a strong growth-suppressive effect, as previously documented by us and others [19, 69, 70]. The observed activation of HALLMARK_E2F_TARGETS signature in LNCaP cells and suppression in the RWPE-1-AR in presence of DHT supports this hypothesis.

The magnitude of chromatin remodeling upon AR activation was greater in RWPE-1-AR cells than in LNCaP cells, despite both being androgen-responsive. At first glance this difference may seem unexpected, but it likely reflects the distinct culture conditions of these models. LNCaP cells are maintained in serum-supplemented medium, which contains androgens, whereas RWPE-1-AR cells are grown in serum-free medium. Although the bovine pituitary extract supplement in RWPE-1 may contain trace androgen levels, the growth characteristics of RWPE-1-AR in this medium [19] suggest that these cells are effectively androgen-naïve at the start of stimulation.

Previous studies have shown that steroid receptor activation primarily increases contacts between pre-existing chromatin loops and rarely generates new loops [15, 71]. This likely explains chromatin accessibility changes in LNCaP cells, where ARBSs are already established. In contrast, RWPE-1-AR exhibit minimal prior AR occupancy, and their high AR expression appears to drive binding at novel genomic locations, enabling formation of new regulatory connections. This mechanism is consistent with the markedly higher number of DHT-sensitive DAPs in RWPE-1-AR compared to LNCaP cell lines.

AR binding is known to be modulated by TFs such as FOXA1 and HOXB13 in PC [50, 53], and these factors were found to be enriched at DSO-DAPs of LNCaP-pcDNA3.1 and LNCaP-ARhi cells. In contrast, RWPE-1-AR cells exhibited a distinct set of AR co-regulators. Notably, the AP-1 factor binding motif was the second most enriched at RWPE-1-AR DSO-DAPs, present in nearly half of these regions. Tang *et al*. (2022) recently reported that AP-1 factors play a critical role in the SCL subtype of CRPC [16]. Both RWPE-1 lines showed greater enrichment at SCL subtype-specific regions compared to LNCaP cell lines, which displayed more enrichment at AR subtype-specific regions. This is consistent with our previous results showing that RWPE-1 cells have gene expression signature similar to luminal progenitor cells such as club and hillock cells [19]. Furthermore, AR activation in RWPE-1-AR shifted the open chromatin landscape toward the signature of AR-subtype, accompanied by a reduction in SCL subtype-specific signals.

AR activation in NPEC cells appeared to promote chromatin compaction at GRHL1/2 binding sites, which overlapped with 39–67% of the closing regions. GRHL2 is a transcriptional repressor that remodels chromatin at its target genes by recruiting polycomb repressive complex proteins (PcG). It has been linked to keratinocyte differentiation, where it regulates genes involved in tight junction formation [72]. The observed decrease in accessibility at GRHL2 binding sites may result from the chromatin-repressing function of this TF. Of the two genes, *GRHL1* is more highly expressed in RWPE-1-AR, whereas *GRHL2* shows slightly higher expression in LNCaP cell lines. Both genes are induced in both cell lines after DHT stimulation.

To evaluate the malignant aspects of the chromatin responses to androgens, we examined the enrichment of ARBSs from PC cohorts reported by Pomerantz *et al*. (2015), Pomerantz *et al*. (2020), and Stelloo *et al*. (2018) [50, 53, 55]. Our previous work demonstrated that genes more highly expressed in normal samples associated with normal tissue-specific ARBS within 50 kb (N-ARBS) are more highly expressed in RWPE-1-AR compared to LNCaP-pcDNA3.1 and -ARhi [19]. Conversely genes more highly expressed in tumor samples and linked to tumor-specific ARBS within 50 kb (T-ARBS) show higher expression in the LNCaP lines[19]. At the chromatin level, RWPE-1-AR DSO-DAPs detected in 10 or 100 versus 0 nM DHT were moderately enriched for N-ARBS, whereas LNCaP-ARhi DSO-DAPs showed higher enrichment for T-ARBS, consistent with the previous RNA-seq analysis.

Lastly, because AR-regulated gene expression has been primarily characterized in PC cells, we integrated chromatin accessibility data with differential gene expression to identify NPEC-specific candidate AR target genes. We selected *CDKN1C* for further validation, as it may explain the growth-inhibitory effect of DHT previously observed in our model [19]. *CDKN1C*/p57 was induced by DHT in RWPE-1-AR, but not in the LNCaP lines. Several studies have documented *CDKN1C* regulation via GRE at the promoter region [73, 74]. We found that one RWPE-1-AR DSO-DAP located in the last intron of *KCNQ1* gene (depicted in Supplementary Fig. S6D [75]) overlaps with a candidate *CDKN1C* enhancer [76], suggesting functional enhancer activity in NPEC cells. miR-21 has also been shown to downregulate p57 in PC[77]. Notably, miR-21 has been reported as a direct AR target gene in PC [78, 79], suggesting that AR-driven miR-21 expression may contribute to CDKN1C/p57 suppression. This mechanism could act in parallel with the reduced enhancer accessibility observed in the LNCaP model.

Taken together, our analysis of chromatin responses to androgen stimulation in nonmalignant and PC cells reveals key differences in AR binding partners and the genes they may regulate in NPEC compared to PC cells. We further confirmed that our NPEC model recapitulates features of benign prostate biopsies, making it a relevant model for studying normal prostate biology in relation to cancer. In addition, we identified several novel candidate AR target genes and validated one of them, *CDKN1C*, as a direct AR target specific to nonmalignant cells. Further studies are needed to elucidate the dynamics of chromatin responses to androgen stimulation and withdrawal in both NPEC and PC cells.

## Supplementary Material

gkag891_Supplemental_Files

## Data Availability

The source code for the analyses in this manuscript is available on GitHub (https://github.com/NykterLab/Androgen-Receptor-Drives-Divergent-Chromatin-Accessibility-Programs-in-Benign-and-Malignant-Prostate) and Zenodo (https://zenodo.org/records/21699746). Raw sequencing data, BigWig files, background-corrected ATAC-signal tables, and normalized/raw expression tables are deposited in ArrayExpress database: E-MTAB-15731 (ATAC-seq) and E-MTAB-15758 (RNA-seq). Raw qPCR data and original Western blot films are available on Zenodo (https://doi.org/10.5281/zenodo.17093003).
