# Supplementary material for: Androgen receptor drives divergent chromatin accessibility programs in benign and malignant prostate epithelial cells
Source: Nucleic Acids Res. 2026 Sep 16;54(17):gkag891. doi: 10.1093/nar/gkag891 (PMC13578506; doi:10.1093/nar/gkag891)
Supplement: Supplementary file 1 [file gkag891_supplemental_files.zip › Supplementary Figures.pdf]

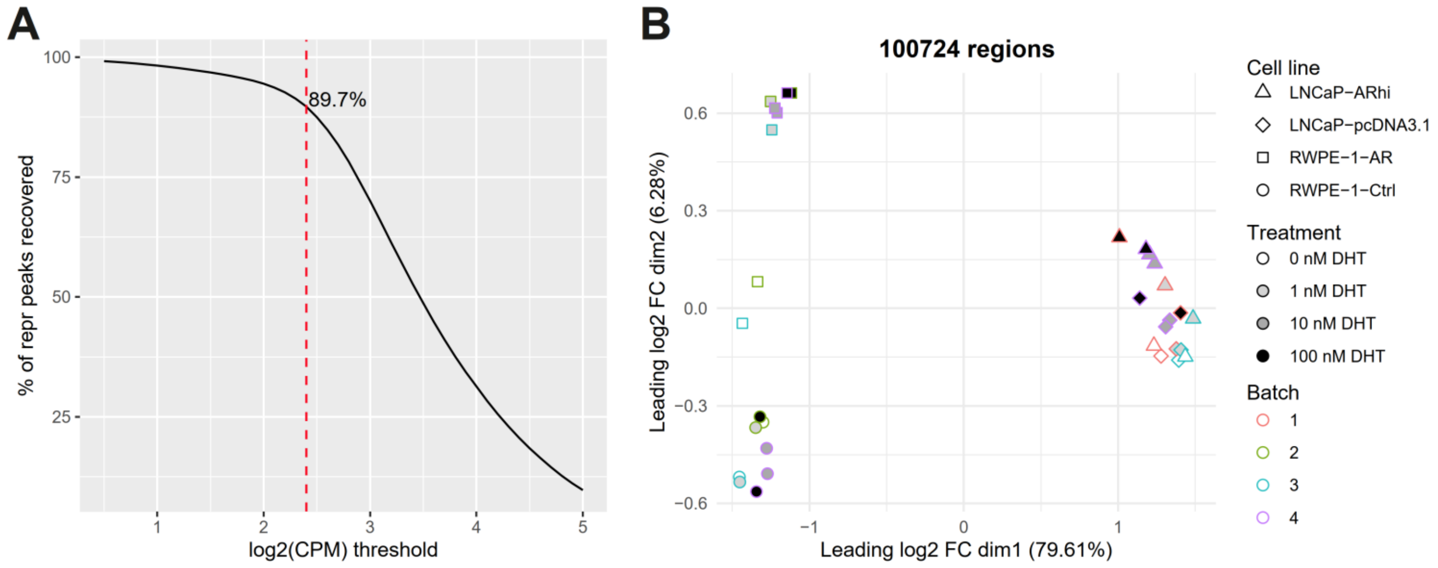

Supplementary Figure S1. A) The proportion of consensus peaks detected as a function log₂(CPM) threshold used. The first elbow is at 2.4 (dashed line). B) Multidimensional scaling (MDS) plot showing first two dimensions for data that was used for the differential accessibility analysis.

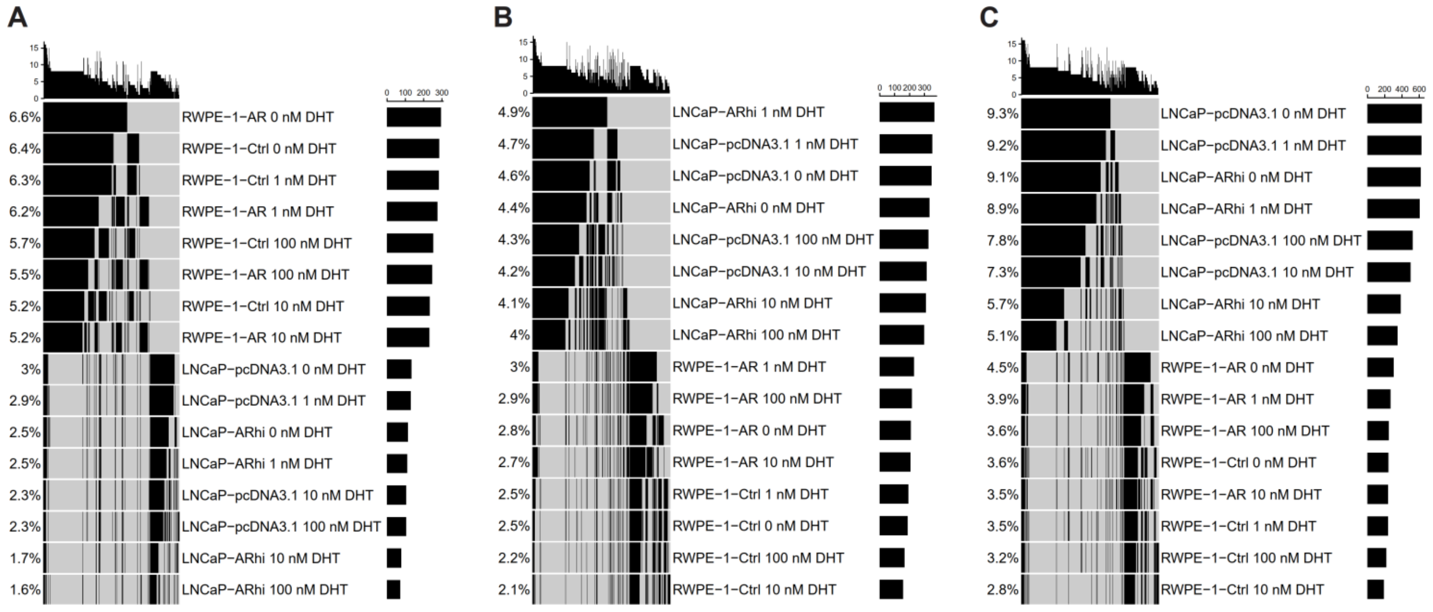

Supplementary Figure S2. Overlap of cell line derived ATAC-seq peaks (with $\log_2$(CPM) > 2.4) and clinical sample ATAC-seq peaks from Tampere PC prostate cancer cohort (Uusi-Mäkelä et al. 2020). A) Overlap with peaks detected only in benign prostate hyperplasia (BPH). B) Overlap with peaks detected only in untreated prostate cancer (PC). C) Overlap with peaks detected only in castration-resistant prostate cancer (CRPC).

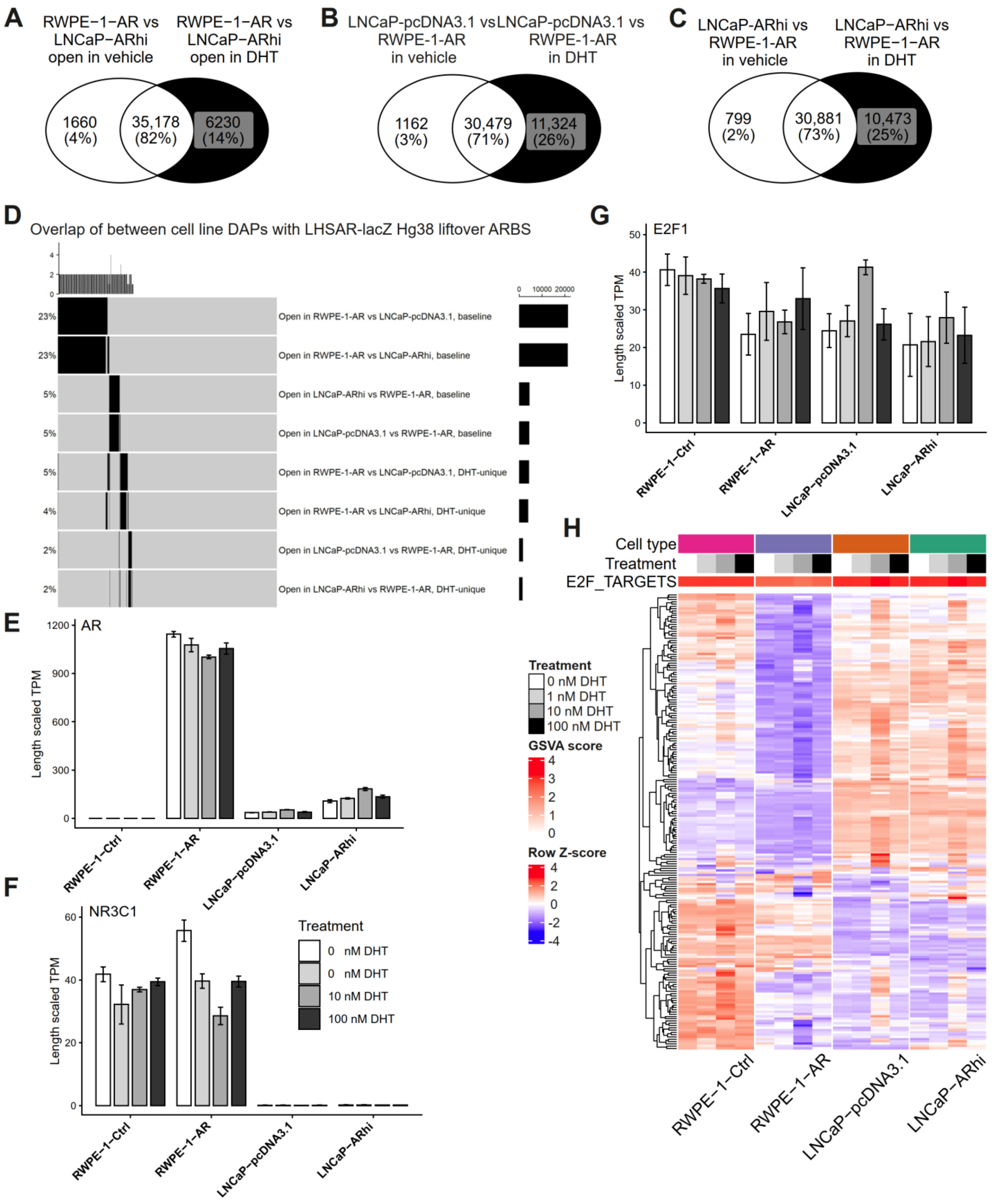

Supplementary Figure S3. The numbers of DAPs between cell lines in the baseline condition or in DHT-treated samples A) in RWPE-1-AR vs LNCaP-ARhi comparison, B) in LNCaP-pcDNA3.1 vs RWPE-1-AR comparison and C) in LNCaP-ARhi vs RWPE-1-AR comparison. D) Overlap of cell type comparison derived DAPs with ARBS from AR expressing PrEC-LHSAR-lacZ control cell line from Pomerantz et al. 2015. Expression of E) AR, F) NR3C1 encoding GR, and G) E2F1 as determined by RNA-seq across the sample set. H) GSVA score and relative expression of HALLMARK_E2F_TARGETS signature across dataset.

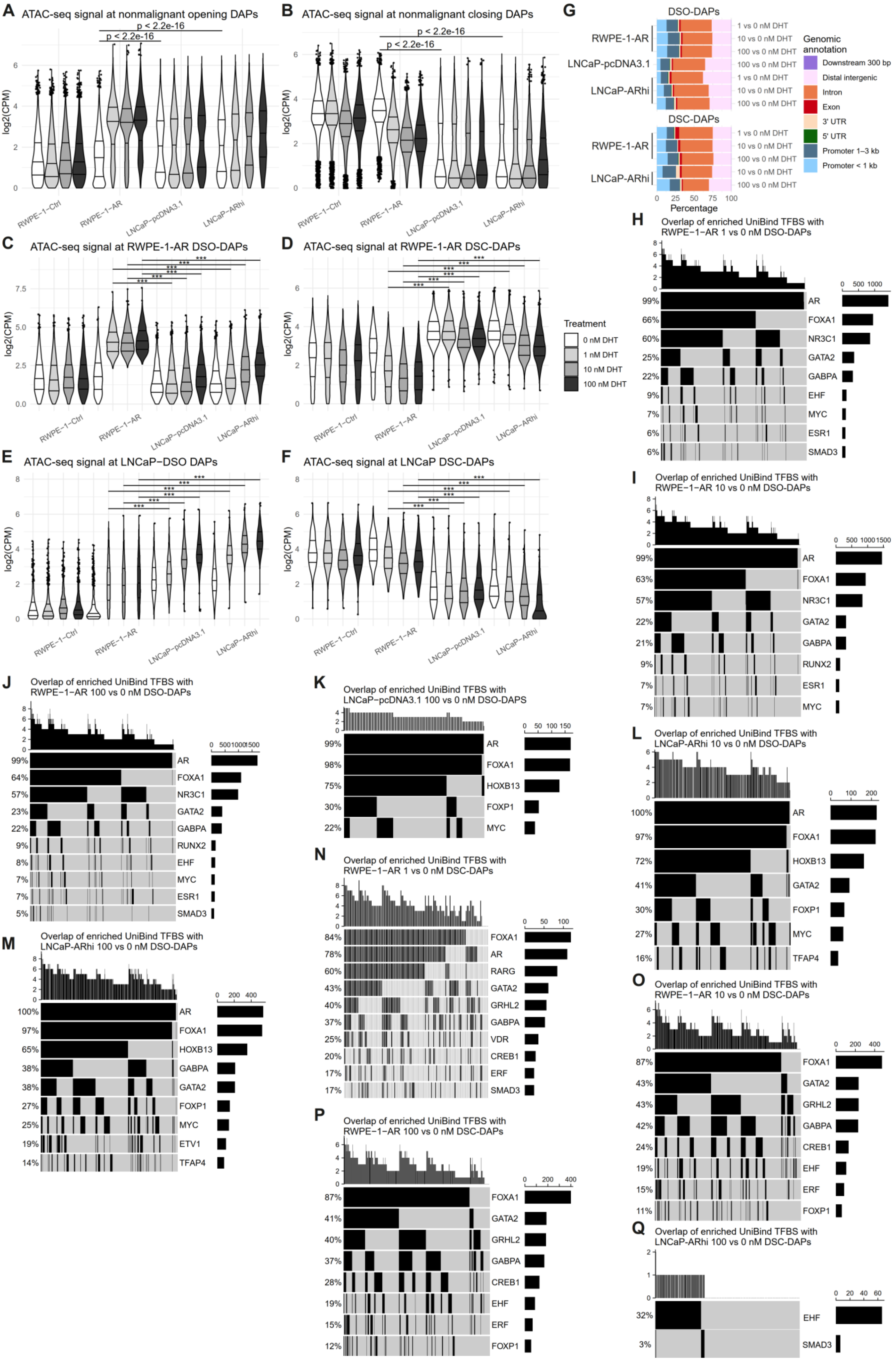
A ATAC-seq signal at nonmalignant opening DAPs
p < 2.2e-16
p < 2.2e-16
log2(CPM)
RWPE-1-Ctrl
RWPE-1-AR
LNCaP-pcDNA3.1
LNCaP-ARhi
B ATAC-seq signal at nonmalignant closing DAPs
p < 2.2e-16
p < 2.2e-16
G
DSO-DAPs
RWPE-1-AR
LNCaP-pcDNA3.1
LNCaP-ARhi
1 vs 0 nM DHT
10 vs 0 nM DHT
100 vs 0 nM DHT
DSC-DAPs
Percentage
Genomic annotation
Downstream 300 bp
Distal intergenic
Intron
Exon
3' UTR
5' UTR
Promoter 1–3 kb
Promoter < 1 kb
C ATAC-seq signal at RWPE-1-AR DSO-DAPs
D ATAC-seq signal at RWPE-1-AR DSC-DAPs
Treatment
0 nM DHT
1 nM DHT
10 nM DHT
100 nM DHT
E ATAC-seq signal at LNCaP−DSO DAPs
F ATAC-seq signal at LNCaP DSC-DAPs
H Overlap of enriched UniBind TFBS with RWPE−1−AR 1 vs 0 nM DSO-DAPs
AR FOXA1 NR3C1 GATA2 GABPA EHF MYC ESR1 SMAD3
I Overlap of enriched UniBind TFBS with RWPE−1−AR 10 vs 0 nM DSO-DAPs
AR FOXA1 NR3C1 GATA2 GABPA RUNX2 ESR1 MYC
J Overlap of enriched UniBind TFBS with RWPE−1−AR 100 vs 0 nM DSO-DAPs
AR FOXA1 NR3C1 GATA2 GABPA RUNX2 EHF MYC ESR1 SMAD3
K Overlap of enriched UniBind TFBS with LNCaP−pcDNA3.1 100 vs 0 nM DSO-DAPS
AR FOXA1 HOXB13 FOXP1 MYC
L Overlap of enriched UniBind TFBS with LNCaP-ARhi 10 vs 0 nM DSO-DAPs
AR FOXA1 HOXB13 GATA2 FOXP1 MYC TFAP4
M Overlap of enriched UniBind TFBS with LNCaP-ARhi 100 vs 0 nM DSO-DAPs
AR FOXA1 HOXB13 GABPA GATA2 FOXP1 MYC ETV1 TFAP4
N Overlap of enriched UniBind TFBS with RWPE−1−AR 1 vs 0 nM DSC-DAPs
FOXA1 AR RARG GATA2 GRHL2 GABPA VDR CREB1 ERF SMAD3
O Overlap of enriched UniBind TFBS with RWPE−1−AR 10 vs 0 nM DSC-DAPs
FOXA1 GATA2 GRHL2 GABPA CREB1 EHF ERF FOXP1
P Overlap of enriched UniBind TFBS with RWPE−1−AR 100 vs 0 nM DSC-DAPs
FOXA1 GATA2 GRHL2 GABPA CREB1 EHF ERF FOXP1
Q Overlap of enriched UniBind TFBS with LNCaP-ARhi 100 vs 0 nM DSC-DAPs
EHF SMAD3

Supplementary Figure S4. TMM-CPM normalized ATAC-seq signal strength distributions at A) Cancerous DHT-opening (padj. < 0.05 and log_2(fold change) > 1) DAPs (the overlap to nonmalignant DHT-opening DAPs removed). B) Cancer DHT-closing (padj. < 0.05 and log_2(fold change) < -1) DAPs (overlap to nonmalignant DHT-closing DAPs removed) ATAC-seq signal strength distributions at C) RWPE-1-AR DSO-DAPs. D) RWPE-1-AR DSC-DAPs E) LNCaP DSO-DAPs F) LNCaP DSC-DAPs. See methods for specifics of the filtering for the cell type-associated, DHT-sensitive (DSO / DSC)-DAPs. G) Distribution of genomic annotations of DS-DAPs. H-Q) Overlaps of the UniBind TFBSs enriching to DS-DAPs according to GIGGLE (shown in Fig. 4B). TFs with low expression (DESeq2 normalized expression < 100) were omitted from the plots.

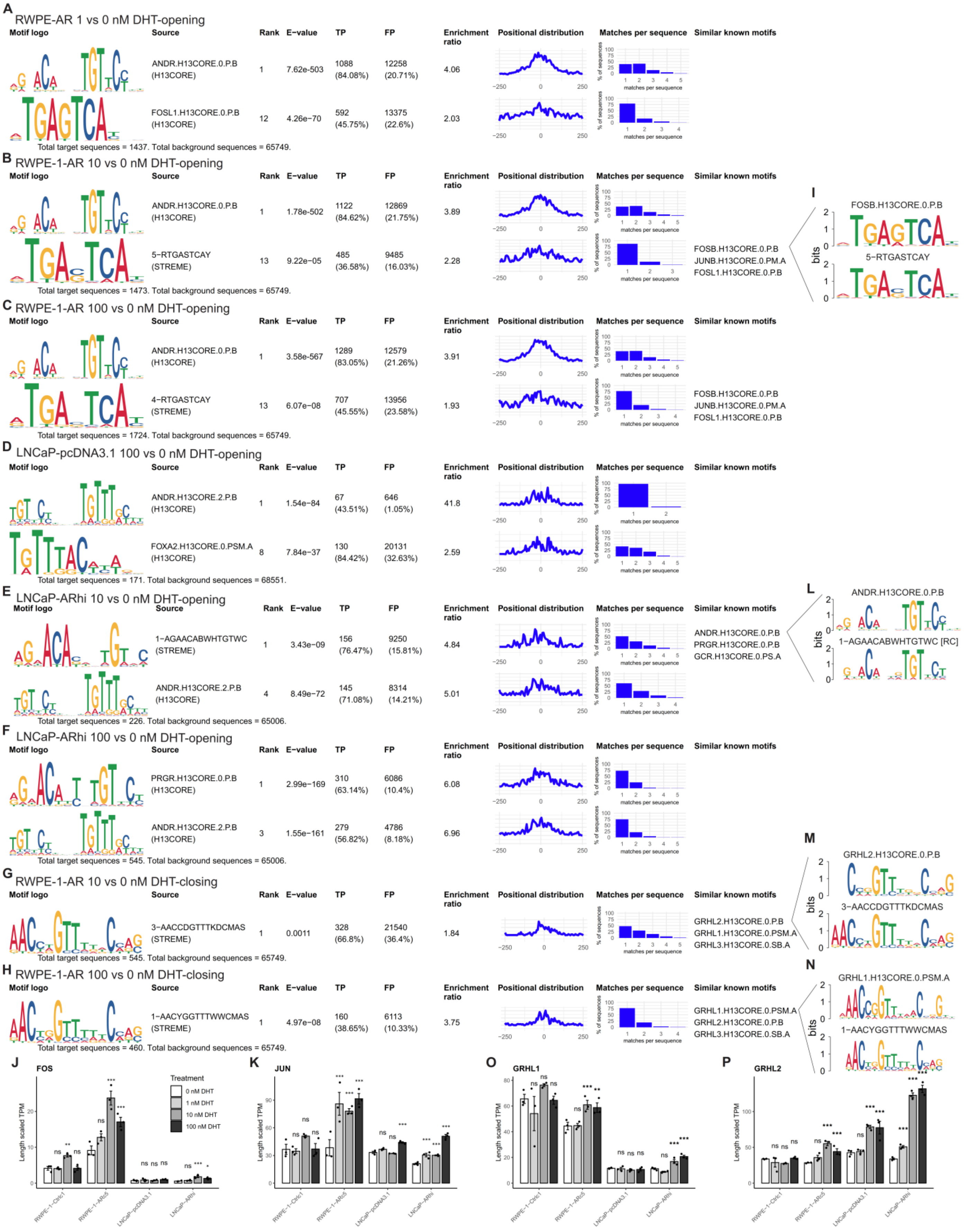

Supplementary Figure S5. XSTREME motif analysis of cell line-associated, DHT-sensitive DAPs. A–H) The most significant motifs (based on rank / E-value) for each comparison are shown. The number of motifs shown for each comparison was selected based on presence of the motif in multiple different comparisons in the same cell line. I, L, M–N) For the *ab initio* motifs, comparison to the most relevant motif is shown on the right. E-values shown are from MEME or STREME for *ab initio* motifs and from SEA

for known motifs, TP number and percentage of significant hits within targets, FP number and percentage of significant motif hits in the background sequences (the reproducible peaks within cell line). Enrichment ratio is the fold enrichment of the motif within target sequences in comparison to background. Expression of J) FOS, K) JUN, O) GRHL1, and P) GRHL2 in the studied cell lines as measured by mRNA-seq. Significance level of * $p < 0.05$, ** $p < 0.01$ *** $p < 0.001$ or ns not significant is from B-H adjusted Wald test.

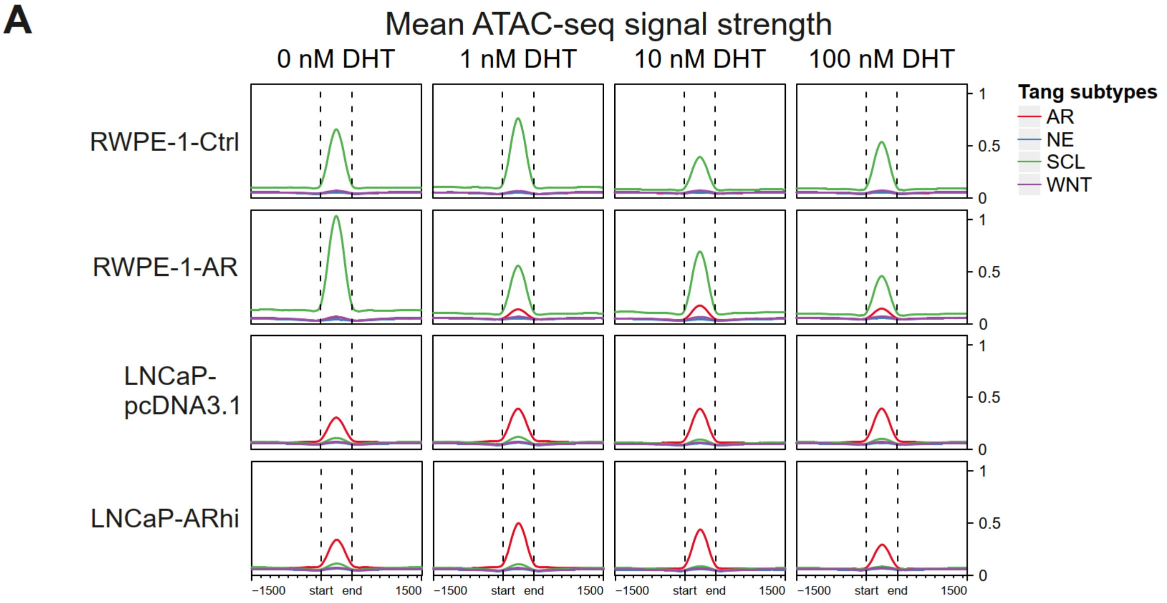

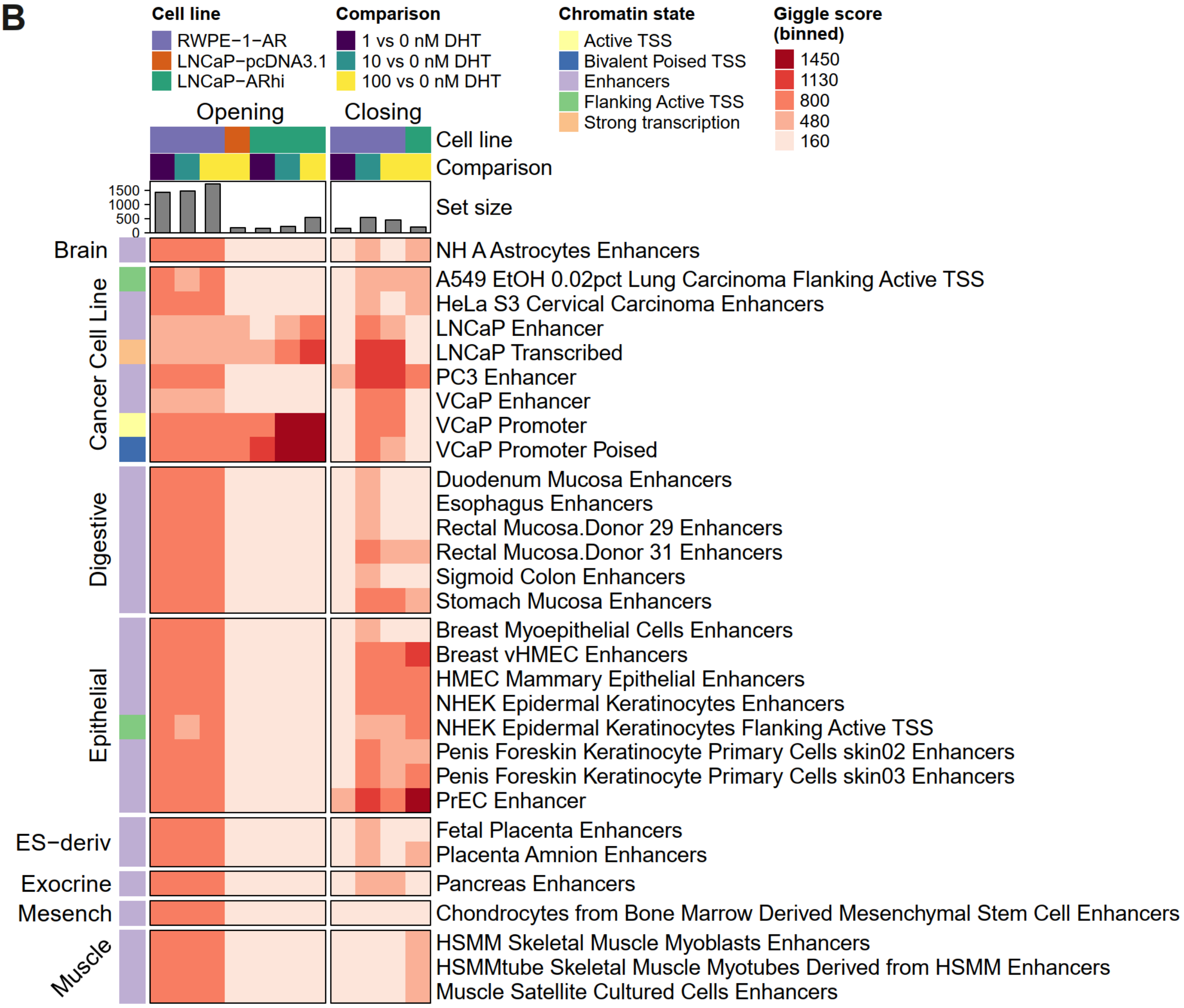

Supplementary Figure S6. A) Mean ATAC-seq signal at the subtype-specific open chromatin regions reported by Tang et al. 2022. B) Enrichment of cell line-associated, DHT-sensitive DAPs to chromatin states from Roadmap Epigenomics database and Gerhauser et al. 2018. 30 categories with the highest enrichment across rows are shown.

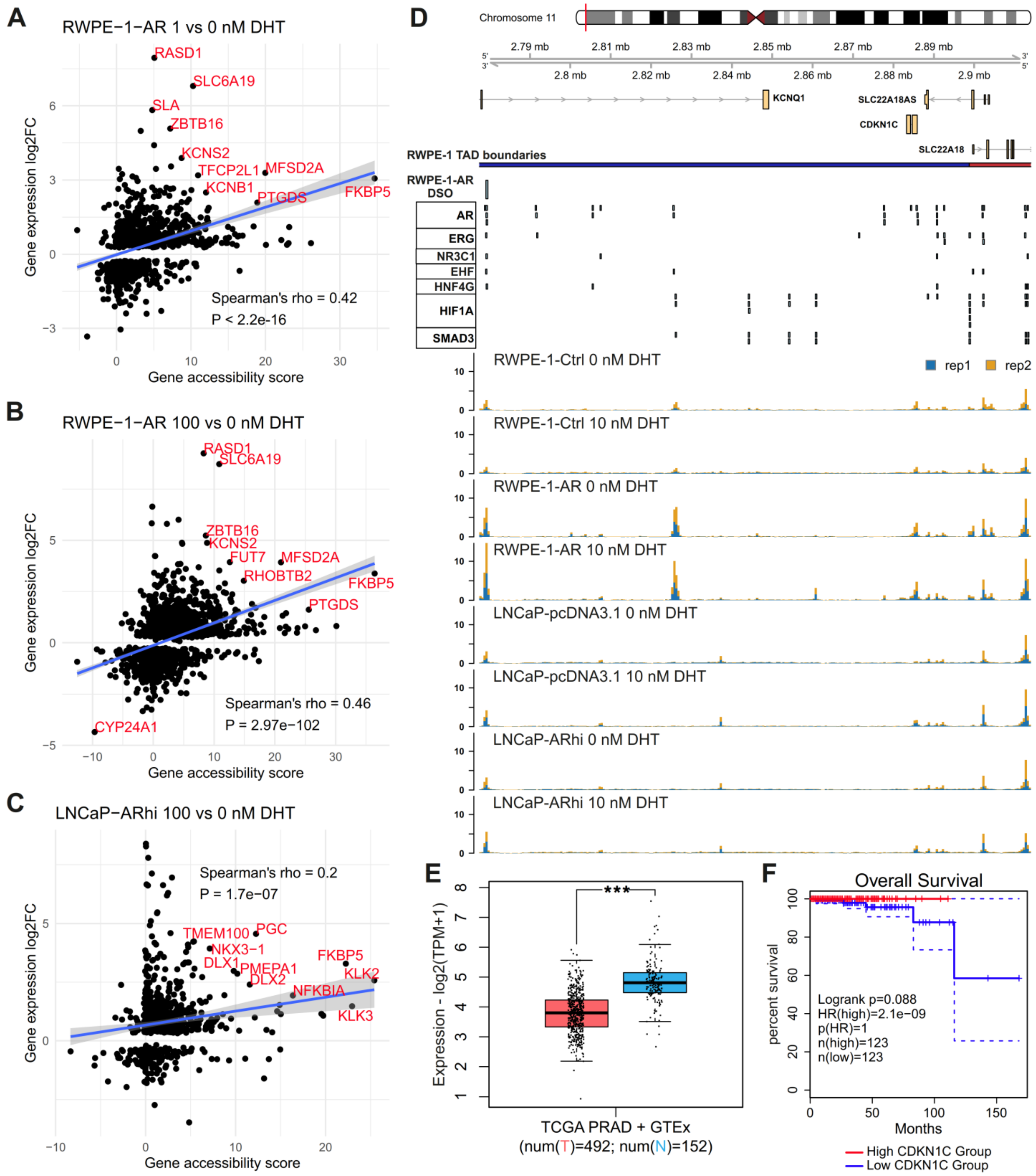

Supplementary Figure S7. Integration of RNA-seq with ATAC-seq. Gene expression log_2(fold change) as a function of gene accessibility score in A) RWPE-1-AR 1 vs 0 nM DHT, B) RWPE-1-AR 100 vs 0 nM DHT, C) LNCaP-ARhi 100 vs 0 nM DHT gene accessibility score was calculated based on DAPs within 250 kb of the gene TSS. Spearman correlation coefficient and p-value for the significance from asymptotic t-approximation. D) *CDKN1C* gene locus with range covering RWPE-1-AR DSO-DAPs within 150 kb of the

TSS of CDKN1C, RWPE-1 TAD boundaries from Rhie et al. 2019 and the relevant TFBS from UniBind robust collection (the ones overlapping with either DSO-DAP). Normalized ATAC-seq signal tracks for 0 and 10 nM DHT in each cell line are shown below. E) The overall survival of patients in CDKN1C high (highest quartile) and CDKN1C low (lowest quartile) groups in the TCGA-PRAD cohort. Data from Gepia2 database. F) Expression of *CDKN1C* in normal tissues (n=152) and tumor tissues n=492 of the TCGA-PRAD cohort + GTEx portal. Data from Gepia2 database. *** indicates p-value < 0.001 from t-test.
